# Identification and Heterologous Expression of the Kendomycin B Biosynthetic Gene Cluster from *Verrucosispora* sp. SCSIO 07399

**DOI:** 10.3390/md19120673

**Published:** 2021-11-26

**Authors:** Jiang Chen, Shanwen Zhang, Yingying Chen, Xinpeng Tian, Yucheng Gu, Jianhua Ju

**Affiliations:** 1 CAS Key Laboratory of Tropical Marine Bio-Resources and Ecology, Guangdong Key Laboratory of Marine Materia Medica, RNAM Center for Marine Microbiology, South China Sea Institute of Oceanology, Chinese Academy of Sciences, 164 West Xingang Road, Guangzhou 510301, China; chenjluck@126.com (J.C.); sherry920111@163.com (S.Z.); chenyingying7788@163.com (Y.C.); xinpengtian@scsio.ac.cn (X.T.); 2 Southern Marine Science and Engineering Guangdong Laboratory (Guangzhou), No.1119 Haibin Road, Guangzhou 511458, China; 3 College of Oceanology, University of Chinese Academy of Sciences, Qingdao 266400, China; 4 Sanya Institute of Oceanology (SCSIO), Zhenzhou Road, Sanya 572000, China; 5 Syngenta Jealott’s Hill International Research Centre, Bracknell, Berkshire RG42 6EY, UK; yucheng.gu@syngenta.com

**Keywords:** kendomycin B, biosynthesis, heterologous expression, *Verrucosispora* sp. SCSIO 07399

## Abstract

*Verrucosispora* sp. SCSIO 07399, a rare marine-derived actinomycete, produces a set of ansamycin-like polyketides kendomycin B–D (**1**–**3**) which possess potent antibacterial activities and moderate tumor cytotoxicity. Structurally, kendomycin B–D contain a unique aliphatic macrocyclic *ansa* scaffold in which the highly substituted pyran ring is connected to the quinone moiety. In this work, a type I/type III polyketide synthase (PKS) hybrid biosynthetic gene cluster coding for assembly of kendomycin B (*kmy*), and covering 33 open reading frames, was identified from *Verrucosispora* sp. SCSIO 07399. The *kmy* cluster was found to be essential for kendomycin B biosynthesis as verified by gene disruption and heterologous expression. Correspondingly, a biosynthetic pathway was proposed based on bioinformatics, cluster alignments, and previous research. Additionally, the role of type III PKS for generating the precursor unit 3,5-dihydroxybenzoic acid (3,5-DHBA) was demonstrated by chemical complementation, and type I PKS executed the polyketide chain elongation. The *kmy* cluster was found to contain a positive regulatory gene *kmy4* whose regulatory effect was identified using real-time quantitative PCR (RT-qPCR). These advances shed important new insights into kendomycin B biosynthesis and help to set the foundation for further research aimed at understanding and exploiting the carbacylic *ansa* scaffold.

## 1. Introduction

The kendomycins are ansamycin-type polyketides possessing an all-carbon macrocyclic *ansa* skeleton characterized by a highly substituted pyran ring and a quinone methide chromophore. In addition to their complex and unique structures, members of the compound class also display remarkable antibacterial, anti-osteoporotic, and anticancer activities; they show great promise as the drug leads for a wide array of clinical applications [1]. By virtue of these properties, the kendomycin class of natural products has long held the interest of the medicinal and synthetic organic communities. To date, the antibacterial and cytotoxic mechanisms of kendomycin action have been widely investigated to reveal an assortment of specific mechanisms of action (MOAs); proteasome impairment and modulation of cation processing account for two of the more dominant and important MOAs demonstrated by the kendomycins [2,3,4]. Not surprisingly, several total syntheses of kendomycin, the first in class member of the family, have been reported and its biosynthesis investigated [1,5,6]. Although chemists and biologists have applied different strategies, both have been challenged by the question of how best to generate the 18-membered carbocycle efficiently. In addition, biologists have long pondered the biosynthetic chronology and enzymology of the post-modifications, underlying the intricate cyclization reactions necessary to attain the complete natural product.

Kendomycin B–D (**1–3**), new analogues of kendomycin, have been produced by *Verrucosispora* sp. SCSIO 07399 [7]. Among these more recently noted members of the class, kendomycin B has been confirmed as a bonafide natural product; kendomycin C and D are both C20-mercapto substituted derivatives of kendomycin B (Figure S1). They are all comprised of a non-methylated quinone moiety which is distinguished from the previously reported kendomycin. Likewise, we have found that kendomycin B shows potent activity against Gram-positive bacteria and moderate cytotoxicity to several human cancer cell lines [7]. Continued biosynthetic interests in the kendomycins revolve around the construction of the macrocarbacyclic *ansa* structure. Here we disclose the: (i) identification and heterologous expression of the biosynthetic gene cluster for kendomycin B (*kmy*); (ii) characterization of a proposed biosynthetic pathway for kendomycin B; and **iii**) exploration of the regulatory role/s of the putative regulatory gene *kmy4*.

## 2. Results and Discussion

### 2.1. Identification of Kendomycin B Biosynthetic Gene Cluster (BGC)

**Figure 1:**
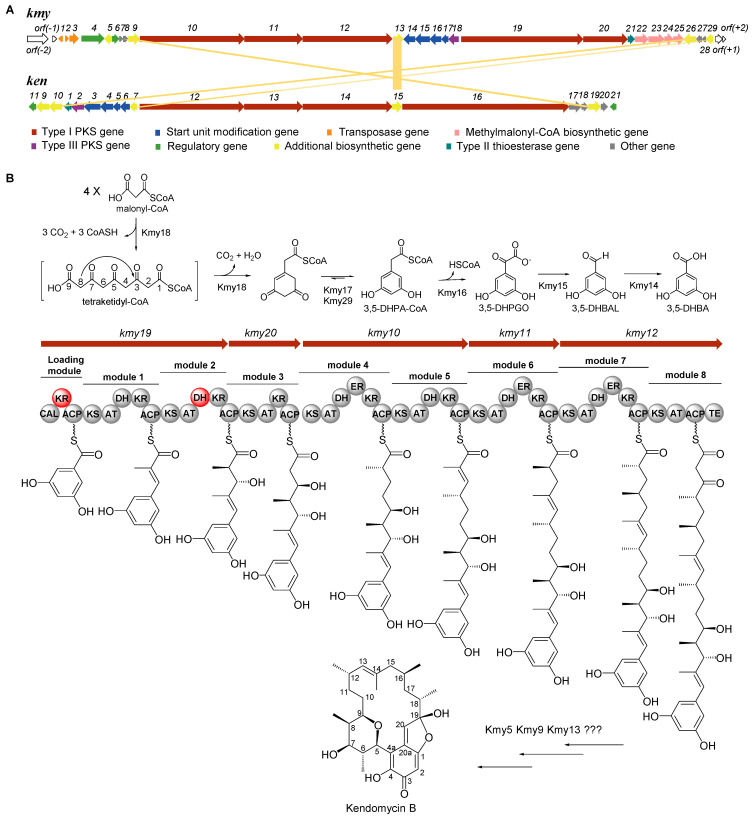
(**A**). Comparative analysis of the *kmy* cluster from *Verrucosispora* sp. SCSIO 07399 in this study and the *ken* cluster from previously reported *Streptomyces violaceoruber* strain 3844–33C. (**B**). The proposed type I/type III hybrid PKS assembly line of kendomycin B with inactivated domains marked in red. KS: ketoacyl synthase; AT: acyl transferase; ACP: acyl carrier protein; DH: dehydratase; KR: ketoreductase; and ER: enoyl reductase, TE: thioesterase.

Whole genome sequencing of *Verrucosispora* sp. SCSIO 07399 was carried out using 2nd generation Illumina HiSeq and 3rd generation PacBio sequencing technologies. The genomic data was analyzed by antiSMASH, which revealed a type I/type III hybrid PKS gene cluster with 75% similarity to the previously established kendomycin cluster (*ken*) [1]. The newly identified cluster contained about 80 kb of information encoding 33 open reading frames (ORF) as shown in Figure 1A. Functional annotation of the genes contained within the kendomycin B BGC (*kmy*) (Table 1) showed high similarity with those of the kendomycin cluster (*ken*) previously reported by Rolf Müller [1]. The main difference between the *kmy* and *ken* clusters is that the *kmy* cluster lacks a methyltransferase gene thought to provide a *para*-methyl moiety of the starter unit; this distinction is accordingly reflected in the solved structure of kendomycin B (Figure S1). In addition, the *kmy* cluster contains additional transposase genes (*kmy1–3*), a set of methylmalonyl-CoA biosynthetic genes (*kmy22–25*), and a number of functionally unknown genes. For further verification, *kmy11* and *kmy18* encoding the type I and type III PKSs were individually inactivated by PCR-targeting technology. The resulting mutant strains were identified and isolated based on kanamycin sensitivity and apramycin resistance phenotypes and genotypically confirmed via PCR (Figures S2 and S3). The purified mutants were fermented and their metabolites were analyzed by HPLC (Figure S4). As hypothesized, neither the ∆*kmy11* nor the ∆*kmy18* mutant produced kendomycin B thus confirming the indispensability of the intact *kmy* cluster for kendomycin B biosynthesis. The *kmy* cluster was submitted to GenBank and the accession number was MZ222135.

### 2.2. Determination of the Boundaries of Kendomycin B BGC 

Bioinformatic analyses suggested early on that the junctions *orf(-2)*-*orf(-1)* and *orf(+1)*-*orf(+2)* flanked either side of the *kmy* cluster. The *orf(-2)*, encoding a DUF262 domain-containing protein, has no homologous counterparts in the *ken* cluster whereas *orf(-1)*, *orf(+1),* and *orf(+2)* all encode proteins for which clear functions are not known. To unambiguously determine the *kmy* cluster boundaries, gene disruption experiments were carried out. The *orf(-2)*-*orf(-1)*, along with three adjacent transposase genes (*kmy1–3*), were coordinately disrupted; the resulting mutant retained wild-type levels of kendomycin B production (Figures S4 and S5), revealing that none of the knocked out genes play a role in kendomycin B biosynthesis. Conversely, joint inactivation of the downstream boundary genes *kmy27*-*orf(+2)* was found to dramatically decrease the yield of kendomycin B; this also was the case upon *kmy29* gene inactivation (Figures S4, S6 and S7). Notably, Kmy29 is a putative enoyl-CoA hydratase/isomerase, whose homolog Ken7 cooperates with another enoyl-CoA hydratase/isomerase Ken3 to enable aromatization after condensing four molecules of malonyl-CoA (M-CoA) by the type III polyketide synthase Ken2 [1]. In light of this realization, *kmy29* was complemented to the *Verrucosispora* sp. SCSIO 07399/Δ*kmy27*-*orf(+2)* mutant (Figure S8). The recovered ability to generate kendomycin B by *Verrucosispora* sp. SCSIO 07399/Δ*kmy27*-*orf(+2)*:*kmy29* (Figure S4) suggested that *kmy27*, *kmy28*, *orf(+1)*, and *orf(+2)* do not play significant roles in kendomycin B biosynthesis. Additionally, the extra set of methylmalonyl-CoA biosynthetic genes (*kmy22–25*) were also jointly inactivated (Figure S9); their inactivation failed to change kendomycin B production (Figure S4). Consequently, we proposed the presence of compensatory methylmalonyl-CoA biosynthesis genes within the genome of *Verrucosispora* sp. SCSIO 07399, but beyond the *kmy* cluster boundaries. The culmination of these data strongly suggested that *kmy4* and *kmy29* very likely constitute the upstream and downstream boundaries of the *kmy* cluster, respectively.

### 2.3. Verification of the Starter Unit 3,5-DHBA for Kendomycin B

Interestingly, the gene clusters for the kendomycins entail hybridized type I and type III PKS genes; the type III PKS genes are responsible for assembling the starter unit. As reported, a set of genes in the *ken* cluster (*ken2*–*7*, *ken9,* and *ken10*) encode the assembly of the proposed starter unit 2,3,5,6-tetrahydroxy-4-methyl-benzoic acid (2,3,5,6-TH-4-MBA) [1]. We found homologous counterparts of *ken2*–*7* and *ken10* in the *kmy* cluster, but failed to identify any homolog to the methyltransferase gene *ken9*. Among the relevant starter unit-related genes, the PQQ-dependent enzyme encoded by *ken10* was regarded as the candidate for hydroxylation of the *ortho*-position of the starter unit. However, the inactivation of the putative *ken10* homolog, *kmy26*, failed to impact kendomycin B production, thus refuting the presumed importance of Kmy26 in carrying out *ortho*-hydroxylation chemistry (Figures S4 and S10). Accordingly, we proposed that the *kmy*-associated PQQ-dependent enzyme is not involved in starter unit construction. Instead, we posit that 3,5-dihydroxybenzoic acid (3,5-DHBA) is a more likely starter unit for kendomycin B. To investigate this hypothesis, chemical complementation using 3,5-DHBA was performed with the type III PKS gene mutant strain *Verrucosispora* sp. SCSIO 07399/Δ*kmy18*. Consistent with our hypothesis, HPLC analyses indicated that supplementation of the *Δkmy18* mutant strain with 3,5-DHBA could indeed restore the production of kendomycin B (Figure S11). Informed by these findings, a revised biosynthetic pathway incorporating elements of previous findings [8,9] is shown in Figure 1B. The type III PKS Kmy18, with 72.8% similarity to Ken2, is envisioned to transform four M-CoAs into 3,5-dihydroxyphenylacetyl-CoA (3,5-DHPA-CoA). This process is facilitated by two enoyl-CoA hydratase/isomerases Kmy17 and Kmy29. The dioxygenase Kmy16 (Ken4 homolog), benzoylformate decarboxylase Kmy15 (Ken5 homolog), and benzaldehyde dehydrogenase Kmy14 (Ken 6 homolog) then convert 3,5-DHPA-CoA to 3,5-DHBA, the bonafide starter unit for kendomycin B assembly.

### 2.4. Analysis of Type I PKS and Tailoring Genes for Polyketide Skeleton Assembly and Cyclization

Structurally speaking, eight condensation steps are required to generate the polyketide skeleton of kendomycin B. Unsurprisingly, eight extension modules were found to be distributed in five open reading frames (ORFs: *kmy10*, *kmy11*, *kmy12*, *kmy19,* and *kmy20*). On the basis of sequence alignments (Figure S12), we anticipated that the acyl-CoA ligase (CAL) domain located in the loading module is responsible for activating the 3,5-DHBA starter unit and binding it to the acyl carrier protein (ACP) domain. Acyltransferase (AT) domains within assembly modules were identified as falling into two general classifications; AT_1_, AT_2_, AT_4_, AT_5_, AT_6_, and AT_7_ all belong to the methylmalonyl AT class which possesses the characteristic YASH motif, whereas AT_3_ and AT_8_ are malonyl AT members distinguished by a conserved HAFH motif able to exclusively select malonate extenders [10]. Extension units are expected to be modified by ketoreductase (KR), dehydratase (DH), and enoyl reductase (ER) domains following their incorporation into the standard PKS assembly line. Significantly, mutations within the conserved motif and residues appear to render the KR of the loading domain unfunctional. Other KR domains could be classified as either A1- or B1-type. KR_1_, KR_2_, and KR_4_-KR_7_ all with the LDD motif but lacking a P residue in their respective catalytic regions were assigned as B1-type KRs. KR_3_ proved to be an A1-type KR having the characteristic W residue but no LDD motif and no H residue [10]. Principally, A- and B-type KRs reduce ketone substrates to l-hydroxy and d-hydroxy intermediates, respectively. The stereochemistry of the KR product subsequently impacts the configuration of olefinic DH domain products. Typically, the l-hydroxy substrate is converted to the *Z*-olefin and the d-hydroxy substrate (of the DH domain) affords the *E*-olefin [11,12]. The highly conserved motif (LxxHxxxGxxxxP) [12] is absent in the DH_2_ domain, leading to DH_2_ inactivation and subsequent retention of the C7-OH moiety. We envisioned that the polyketide chain undergoes release by the TE (thioesterase) domain which was highly homologous to macrocycle-forming TEs such as the DEBS_TE from the cluster for 6-deoxyerythronolide biosynthesis (Figures S13 and S14). We proposed that the TE domain may cyclize the polyketide backbone to stabilize the terminal keto moiety which is ordinarily extremely prone to decarboxylation by virtue of its β-keto acid form [13]. Another possible scenario entails the loss of cyclization function by the TE domain leading to retention of the free β-keto acid which may be protected by unknown proteins. 

The post-PKS tailoring chemistry necessary to obtain kendomycin B involves a complicated macrocyclization of C20 and C20a, an aldol condensation between the C19 carbonyl and C1 hydroxy, and the generation of the pyran ring by an addition reaction of the C5 vinyl and C9 hydroxyl moieties to ultimately afford the unique aliphatic *ansa* system [1]. As noted above, we proposed the intricate cyclization procedure probably involves multiple enzymes. A gene cluster search revealed two flavin-dependent enzymes of interest: FAD-dependent oxidoreductase Kmy9 and FAD-dependent monooxygenase Kmy13. Sequence alignment and phylogenetic analyses (Figures S15 and S16) revealed that Kmy13 is a class A flavoprotein monooxygenase containing the typical GxGxxG fingerprint used to bind the ADP-part of FAD [14,15]. As reviewed, FAD-dependent monooxygenases are very versatile and can catalyze diverse reactions such as the Baeyer–Villiger oxidation, epoxidation, hydroxylation, halogenation, and sulfoxidation. These enzymes are grouped into six subclasses A–F, based on sequence similarities and structural features. The class A monooxygenases usually carry out aromatic ring hydroxylation at either the *ortho*- or *para*-position [14,15]. Thus, Kmy13 likely catalyzes the *ortho*-hydroxylation which, in all likelihood, precedes quinone installation; we envision that the presence of the quinone vastly facilitates subsequent cyclizations en route to kendomycin B. Given the putative role of redox chemistry in the proposed cyclizations, the FAD-dependent oxidoreductase Kmy9 and another NAD(P)-dependent oxidoreductase, Kmy5, may prove vital to establishing the polycyclic backbone of kendomycin B. These proposals are roughly the same as the previous report in which Ken15 (FAD-dependent monooxygenase), Ken17 (lipase), and Ken19 (FAD-dependent oxidoreductase) were highly expected to construct the macrocyclic scaffold of kendomycin [1]. Further details about this cyclization chemistry are forthcoming and will be reported in due course.

### 2.5. Characterization of a Positive Regulatory Genes

The *kmy* cluster is equipped with two regulatory genes, *kmy4* and *kmy6,* at the upstream region. BLAST (Basic Local Alignment Search Tool) assigned Kmy4 as a LuxR family transcriptional regulator. More precisely, phylogenetic analysis (Figure S17) classified Kmy4 into the group of LAL-type regulators (Large ATP-binding regulators of the LuxR family), which team up with pathway-specific transcriptional activators PikD [16] and GdmR1 [17]. Correspondingly, multiple sequence alignments of Kmy4 and other LAL-type regulators (Figure S18) revealed the characteristic Walker A motif (GxxxxGK[S/T]) for ATP/GTP-binding at the Kmy4 N-terminal region and a characteristic helix-turn-helix (HTH) DNA-binding motif at the C-terminal region [18,19]. Importantly, genetic inactivation of *kmy4* abolished kendomycin B production; kenomycin B production could, as predicted, be recovered by gene complementation. Similar experiments with *kmy6* revealed no difference in kendomycin biosynthesis (Figures S19–S22); metabolite titers remained unchanged regardless of Kmy6 activity. Accordingly, Kmy4 was assigned to be a pivotal positive regulator of kendomycin B production; and Kmy6 may mediate the expression of multidrug efflux pumps like most AcrR family regulators [20,21], but its precise function remains unknown. 

**Figure 2:**
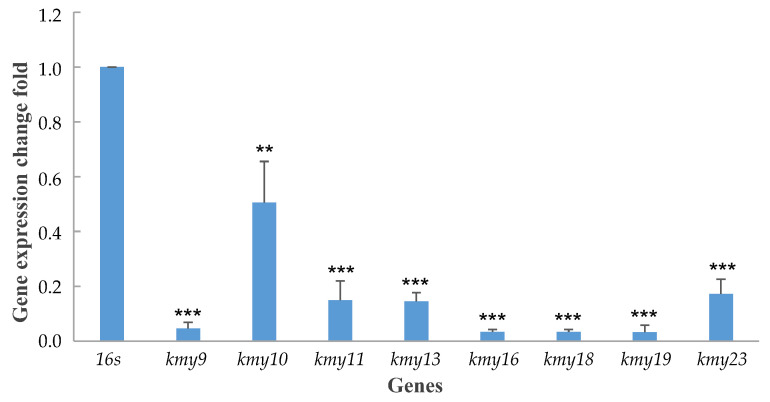
Relative change fold of eight kendomycin B biosynthetic genes in transcriptional regulator gene mutant strain *Verrucosispora* sp. SCSIO 07399/Δ*kmy4*, comparing with wild-type strain *Verrucosispora* sp. SCSIO 07399. *p* value < 0.001 was marked as ***, *p* value < 0.01 was marked as **.

To expand upon the role of Kmy4 in kendomycin B biosynthesis, we next investigated the transcriptional effects of Kmy4. Transcription levels for genes within the *kmy* cluster in mutant strain Δ*kmy4* were contrasted with those of the wild-type strain using real-time qPCR methodology. We specifically selected eight representative genes for the survey; these included type I and type III PKS genes, starter unit biosynthetic genes, and post-PKS tailoring genes, all specifically regulated by different promoters. The mRNA quantitation experiment revealed dramatic decreases in gene transcription levels following *kmy4* inactivation (Figure 2). Especially hard hit by the loss of *kmy4* were starter unit assembly genes *kmy16* and *kmy18* (type III PKS), a polyketide assembly gene *kmy19,* and a post-PKS modification gene *kmy9*. These findings not only validated our assignment of Kmy4 as a positive regulator of kendomycin B biosynthesis, but they also help to explain the underpinnings of Kmy4 regulatory function. 

### 2.6. Heterologous Expression of Kendomycin B BGC 

**Figure 3:**
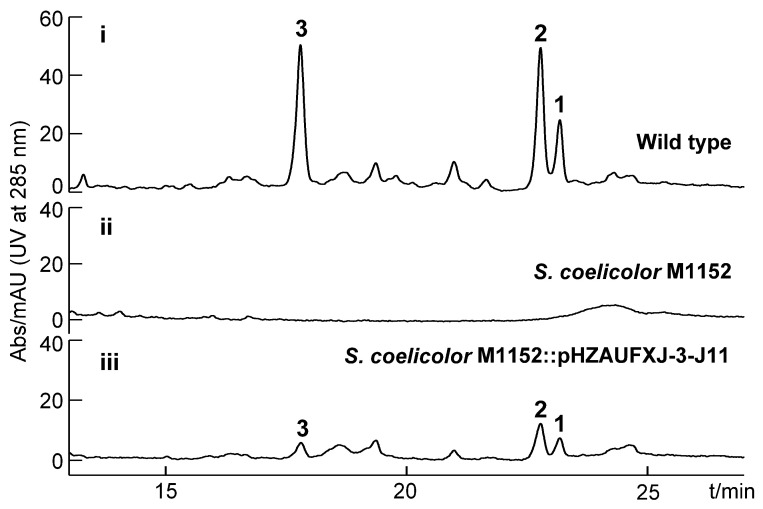
HPLC analysis of fermentation broths from (**i**) the wild-type strain *Verrucosispora* sp. SCSIO 07399 from which *kmy* cluster was identified; (**ii**) host strain *Streptomyces coelicolor* M1152 devoid of *kmy* cluster; and (**iii**) heterologous expression system involving *kmy* cluster expression in *S. coelicolor* (strain designation = *Streptomyces coelicolor* M1152:pHZAUFXJ-3-J11).

Heterologous expression of natural product biosynthetic gene clusters has been extensively employed in the fields of combinatorial biosynthesis and metabolic engineering. Heterologous expression strategies can not only boost the production of promising natural products and optimize genetic operating systems, but they can also provide improved access to new analogs and activate otherwise cryptic/silent gene clusters en route to novel biomolecules [22]. Notably, *Verrucosispora* sp. SCSIO 07399 is a marine-derived rare actinomycete with poor sporulation ability and slow growth; both deficiencies impair stable production of kendomycin B–D. To improve our access to these metabolites, we sought to heterologously express the *kmy* cluster in *Streptomyces coelicolor* M1152. We screened a recombinant plasmid pHZAUFXJ-3-J11 carrying the complete *kmy* BGC from the bacterial artificial chromosome (BAC) library of *Verrucosispora* sp. SCSIO 07399. The pHZAUFXJ-3-J11 plasmid was then introduced into the heterologous host by triparental intergeneric conjugation, affording *S. coelicolor* M1152:pHZAUFXJ-3-J11. Importantly, validation of the desired cluster integration and fidelity within *S. coelicolor* M1152 was readily achieved via PCR and HPLC-based metabolite analyses (Figure S23 and Figure 3). HPLC analysis of the hetero-expression strain fermentation extract revealed that the *kmy* cluster could successfully express in *S. coelicolor* M1152 and stably produce kendomycin B–D, although the yield was substantially reduced by 77.8% relative to the wild-type producer. This reduced yield may be the result of physiological differences between the two species, such as substrate supply, the influence of metabolic branches, and the in vivo environment for the enzymatic reaction. Optimization of fermentation conditions and/or genetic modifications of the *S. coelicolor* M1152:pHZAUFXJ-3-J11 producer will need to be carried out to improve kenodmycin yield. 

## 3. Materials and Methods

### 3.1. General Experimental Procedures

All bacterial strains, plasmids, and primers applied in this study are listed in Tables S1 and S2. *Verrucosispora* sp. SCSIO 07399 isolated from the deep-sea sediment of the South China Sea was the producer of kendomycin B–D in this study. Nine gene mutant strains of *Verrucosispora* sp. SCSIO 07399 and two gene-complemented strains were constructed in this study to explore the biosynthesis of kendomycin B. The wild-type, mutant, and complemented strains were all cultivated on a solid modified ATCC 172 medium (soluble starch 20 g/L, glucose 10 g/L, yeast extract 5 g/L, AoBoX casein 5 g/L, CaCO_3_ 19 g/L, crude sea salt 30 g/L, and 2% agar, pH 7.2–7.4) with appropriate antibiotics to grow mycelium and the modified RA medium (1% glucose, 1% maltose extract, 0.5% corn flour, 2% soluble starch, 1% maltose, 3% crude sea salt, and 0.2% CaCO_3_, pH 7.2–7.4) was used for fermentation, the optimum culture temperature was 28 °C. *E. coli* type strains were cultivated using LB (Luria-Bertani) medium (0.5% yeast extract, 1% tryptone, and 1% NaCl, pH 7.0–7.4) at 37 °C.

Antibiotics were used in previously optimized concentrations: 25 μg/mL chloramphenicol (Cml), 50 μg/mL apramycin (Apr), 50 μg/mL kanamycin (Kan), 50 μg/mL trimethoprim (TMP), and 25 μg/mL thiostreptone (Tsr). 

All primers were synthesized by Sangon Biotech (Shanghai) Co. Ltd. (Shanghai, China). All reagents used in this study were purchased from Sigma-Aldrich Company Ltd. (Vienna, Austria), Toronto Research Chemicals Inc. (Toronto, ON, Canada), Sangon Biotech (Shanghai) Co. Ltd. (Shanghai, China), Amatek Scientific Co. Ltd. (Berwyn, Pennsylvania) and Shanghai Macklin Biochemical Co. Ltd. (Shanghai, China).

### 3.2. Genome Sequencing Assembly and DNA Sequence Analysis

The whole genome of *Verrucosispora* sp. SCSIO 07399 was sequenced and assembled by Shanghai Biozeron Technology Co. Ltd using 2nd generation Illumina HiSeq and 3rd generation PacBio sequencing technologies. Online bioinformatics software antiSMASH (https://antismash.secondarymetabolites.org, accessed on 10 November 2021) led to the analysis and annotation of secondary metabolite gene clusters. Gene function predictions were performed using the established online BLAST program (https://blast.ncbi.nlm.nih.gov/Blast.cgi, accessed on 10 November 2021). Domains contained in each gene were identified by PKS/NRPS Analysis Web-site (http://nrps.igs.umaryland.edu, accessed on 10 November 2021). 

### 3.3. Construction of Gene Mutant Strains

According to the REDIRECT protocol [23], 20 functional genes (*orf(-2)*-*kmy3*, *kmy4*, *kmy6*, *kmy11*, *kmy18*, *kmy22–25*, *kmy26, kmy27-orf(+2),* and *kmy29*) of *kmy* cluster from *Verrucosispora* sp. SCSIO 07399 were individually or jointly inactivated using λ-RED recombination technology. Five cosmids 3–6B, 1–8B, 5–9H, 4–11A, and 6–1A were selected for gene disruption. The apramycin resistance gene cassette *oriT/aac(3)IV* was used to partially replace the target gene. During the conjugation process, mutant cosmids were initially transferred into non-methylating *E. coli* ET12567/pUZ8002, then introduced into *Verrucosispora* sp. SCSIO 07399 with the help of the non-transmissible plasmid pUZ8002. The mixture of *E. coli* and actinomycete were spread on the solid modified MS (Murashige and Skoog) medium (2% soy flour, 2% mannitol, and 2% agar, pH 7.2–7.4) containing 20 mM Mg^2+^. After about 18 h incubation at 28 °C, 1 mL ddH_2_O with 25 μL Apr (50 mg/mL) and 25 μL TMP (50 mg/mL) were coated evenly onto the plates, and the plates were then transferred to a 37 °C incubator to generate exconjugants. Double exchange mutant strains, which were apramycin-resistant but kanamycin-sensitive, were identified by resistance screenings and PCR verification. 

### 3.4. Gene Complementation of Δkmy4 and Δkmy27-orf(+2) Mutant Strains

The plasmid pL646ATE-ssaA-BXSE, equipped with the site-specific recombinase φC31 system, was used to complement the *kmy4* and *kmy29* genes to their corresponding mutant strains *Verrucosispora* sp. SCSIO 07399/Δ*kmy4* and Δ*kmy27-orf(+2)*, respectively. The constructed plasmids pL646ATE-ssaA-BXSE-*kmy4* and pL646ATE-ssaA-BXSE-*kmy29* were transferred to Δ*kmy4* and Δ*kmy27-orf(+2)* mutant strains by conjugation mediated by *E. coli* ET12567/pUZ8002, affording complemented strains *Verrucosispora* sp. SCSIO 07399/Δ*kmy4*:*kmy4* and *Verrucosispora* sp. SCSIO 07399/Δ*kmy27-orf(+2)*:*kmy29*.

### 3.5. Small-Scale Fermentation and Analyses

The wild-type strain *Verrucosispora* sp. SCSIO 07399, gene mutant strains, and gene complemented strains were incubated on solid modified ATCC 172 medium with appropriate antibiotics at 37 °C. A portion of mycelium was then inoculated into 50 mL of modified RA medium in a 250 mL flask and cultivated in a 200 rpm shaker at 28 °C for 6–7 days. The culture of each strain was extracted with an equal volume of butanone, organic phases were evaporated to dryness, and the remaining residue redissolved in 500 μL MeOH, 30 μL of which was injected into analytical HPLC for product analysis. The detection wavelengths were 210 nm, 254 nm, 275 nm, 285 nm, 360 nm, and 500 nm. The mobile phase was comprised of solvent A and solvent B; solvent A consisted of ddH_2_O supplemented with 0.1% TFA and solvent B consisted of CH_3_CN supplemented with 0.1% TFA. Samples were eluted using a linear gradient from 5% to 80% solvent B in 20 min, then 80% to 100% solvent B in 1 min, and finally 100% solvent B for 5 min; this complete HPLC process was carried out at a flow rate of 1.0 mL/min.

### 3.6. Chemical Complementation of Type III PKS Gene Mutant Strain Δkmy18

The commercially available 3,5-dihydroxybenzoic acid (3,5-DHBA) powder was dissolved in DMSO at concentration of 10 mg/mL. The type III gene mutant strain *Verrucosispora* sp. SCSIO 07399/Δ*kmy18* was inoculated into 50 mL modified RA medium, and after a 2 d cultivation at 200 rpm agitation at 28 °C, 50 μL of 3,5-DHBA (10 mg/mL) was added and fermentation carried out for another 2 d. Following a 4 d incubation, another 50 μL of the 3,5-DHBA stock solution was added into the fermentation liquid. The complete mixture was fermented for another 3 d (at 200 rpm agitation/shaking) at 28 °C. Following the full 7 d fermentation, the fermentation broth was extracted and metabolites analyzed by HPLC. 

### 3.7. Phylogenetic Analysis and Sequence Alignment

First, multiple sequences were aligned by ClustaW, then the phylogenetic tree of Kmy4 and other LuxR family regulators was constructed by MEGA (Version 5.05) using the neighbor-joining method with Poisson correction model, and the bootstrap value of 1000 replications. All types of LuxR regulators used in phylogenetic analysis are listed as follows: PteF (BAC68119.1) from *Streptomyces avermitilis*, AurJ3M (ACD75765.1) from *Streptomyces aureofuscus*, AmphRIV (AJE39070.1) from *Streptomyces nodosus*, FilF (AKX77828.1) and FilR (AKX77827.1) from *Streptomyces filipinensis*, FscRI (SUP34276.1) from *Streptomyces griseus*, SlnM (AHB62093.1) from *Streptomyces lydicus*, PimM (CAM35468.1) and PimR (CAM35469.1) from *Streptomyces natalensis,* ScnRI (ADX66458.1) from *Streptomyces chattanoogensis*, PikD (AAC68887.1) from *Streptomyces venezuelae*, GdmRI (ABI9379.1) from *Streptomyces hygroscopicus*, TrdH (ADY38540.1) from *Streptomyces* sp. SCSIO 1666, PldR (BAH02275.1) from *Streptomyces platensis*, SlgR2 (CBA11556.1) from *Streptomyces lydicus*, LuxR (P12746.3) from *Vibrio fischeri*, TraR (AAD31600.1) from *Agrobacterium radiobacter* K84, LasR (NP_250121.1) from *Pseudomonas aeruginosa* PAO1, SdiA (AAC08299.1) from *Salmonella enterica*, and SmaR (CAB92554.1) from *Serratia* sp. ATCC 39006.

All thioesterases used in the phylogenetic analysis are listed as follows: Ken14_TE (CAQ52624.1) from *Streptomyces violaceoruber*, DEBS_TE (X62569.1) from *Saccharopolyspora erythraea* NRRL 2338, PICS_TE (AF079138.1) from *Streptomyces venezuelae*, Averm_TE (BAA84479.1) from *Streptomyces avermitilis*, TgaC_TE (ADH04641.1) from *Sorangium cellulosum*, TesB1 (CCP44382.1) from *Mycobacterium tuberculosis* H37Rv, GrsT (AEI41826.1) from *Paenibacillus mucilaginosus* KNP414, MonAX (ANZ52473.1) from *Streptomyces cinnamonensis*, NysE (AAF71777.1) from *Streptomyces noursei* ATCC 11455, NanE (AAP42868.1) from *Streptomyces nanchangensis*, TylO (WP_043472398.1) from Streptomyces, RifR (AAG52991.1) from *Amycolatopsis mediterranei* S699, and K-41A_TE (MT318810.1) from *Streptomyces* sp. SCSIO 01680.

Multiple sequence alignment was carried out using ClustalX 1.83 software and refined with online ESPript 3.0 software (http://espript.ibcp.fr/ESPript/cgi-bin/ESPript.cgi, accessed on 10 November 2021). Conserved motifs and residues were marked manually. 

### 3.8. RNA Extraction of Wild-Type and Δkmy4 Mutant Strains, cDNA Synthesis, and Real-Time Polymerase Chain Reaction

*Verrucosispora* sp. SCSIO 07399 and Δ*kmy4* were inoculated into 50 mL of TSB (Tryptic Soy Broth) medium in a 250 mL flask and cultivated for 2 d in a 28 °C 200 rpm shaker to acquire mycelium. Total RNAs of wild-type and Δ*kmy4* mutant strains were extracted using Trizol reagent (Takara Biotechnology, Japan) from 100 μL mycelium. cDNAs were synthesized by reverse transcriptions (RTs) following the manufacturer’s instructions for the PrimerScriptTM RT reagent kit (Takara Biotechnology, Kusatsu, Japan). Real-time qPCR using ChamQ Universal SYBR qPCR Master Mix (Vazyme Biotech Co., Itd., Nanjing, China) was performed on an ABI Real-Time qPCR Fast System VIIA7. The amplification system of real-time qPCR was optimized, consisting of 1 μL 5-fold diluted cDNA template (about 10 ng/μL), 1.5 μL 30-fold diluted forward and reverse primers mixture (about 0.33 μM), and 2.5 μL qPCR master reagent. The qPCR procedure was started with preincubation at 95 °C for 20 s, followed by 40 cycles of denaturation at 95 °C for 1 s, then annealing and extension at 60 °C for 20 s. 

The expression levels of eight genes (*kmy9*, *kmy10*, *kmy11*, *kmy13*, *kmy16*, *kmy18*, *kmy19*, and *kmy23*) within the *kmy* biosynthetic gene cluster in both wild-type and Δ*kmy4* mutant strains were detected at the transcription level using real-time qPCR and one 16s rRNA as the internal reference. The Ct value of each detected sample was recorded, and corresponding ΔCt was calculated by subtracting the Ct value of the reference gene. Then the ΔCt value of each gene in the Δ*kmy4* mutant subtracted the corresponding ΔCt value of wild-type strain, known as ΔΔCt. The relative expression of each gene was calculated using the equation: relative change fold = 2^(−ΔΔCt). The above assays were all performed in triplicate.

### 3.9. Heterologous Expression of the kmy Gene Cluster

The BAC library was constructed by Genomic Resources Laboratory, Huazhong Agricultural University (http://Gresource.hzau.edu.cn, accessed on 10 November 2021) [24]. Three pairs of primers located in the upstream, middle, and downstream regions of the kendomycin B BGC were used for genomic library screening. A recombinant BAC plasmid, pHZAUFXJ-3-J11 containing the complete *kmy* gene cluster, was selected. According to the triparental conjugation protocol previously described [25], the plasmid pHZAUFXJ-3-J11 was transferred into heterologous host *Streptomyces coelicolor* M1152 with the help of *E. coli* ET12567/pUB307. Exconjugants were screened for the apramycin resistant phenotype and PCR; positive clones were further verified by fermentation. 

## 4. Conclusions

A type I/type III PKS hybrid biosynthetic gene cluster encoding the assembly of kendomycin B (*kmy*) was identified from marine-derived *Verrucosispora* sp. SCSIO 07399. The *kmy* cluster was distinguished from the previously reported kendomycin gene cluster (*ken*) predominantly by its lack of a methyltransferase gene. The essentiality of the *kmy* cluster to kendomycin B construction was verified by ketosynthase (KS) gene disruption and heterologous expression experiments. Guided by bioinformatic analyses and previous research, systematic gene disruptions were carried out, enabling us to determine the *kmy* cluster boundaries and also unveiling a positive regulator Kmy4. Multiple sequence alignments and phylogenetic analyses revealed Kmy4 as a LAL-type LuxR family regulator; its positive regulatory role was further confirmed by RT-qPCR. In addition, we have formulated a cogent biosynthesis of kendomycin B which is initiated by 3,5-DHBA (starter unit) construction by the type III PKS. This component of the proposed biosynthesis was verified by chemical complementation experiments. We also proposed that the polyketide chain produced by the type I PKS assembly line be then subjected to a set of cyclizations leading to the unique carbacylic *ansa* scaffold. We proposed the coordination of a set of oxidoreductases that catalyzed the intricate cyclization sequence. Of these oxidoreductases, the FAD-dependent monooxygenase Kmy13 was regarded as the best candidate for carrying out an important *ortho*-hydroxylation of the aromatic ring. Though highly illuminating, these studies highlight outstanding questions that warrant continued study. Answers to these questions (in progress), along with our findings here, will help to establish the foundation for future combinatorial biosynthetic efforts to diversify and optimize kendomycin structures and potential biomedical applications. 

## Figures and Tables

**Table 1:** Functional annotation of ORFs in the kendomycin B biosynthetic gene cluster.

ORF	Size ^a^	Proposed Function	Closest Homolog, Origin (Protein ID); ID/SI (%)	*ken* Homolog
*orf(-2)*	778	DUF262 domain-containing protein	DUF262 domain-containing protein, *Salinispora arenicola* (WP_028680247.1); 98.2/100	-
*orf(-1)*	95	Hypothetical protein	Hypothetical protein BCD48_40600, *Frankia* sp. BMG5.36 (OHV60767.1); 57.8/47	-
*kmy1*	87	Transposase	Transposase, *Micromonospora marina* (WP_141708283.1); 96.5/100	-
*kmy2*	78	Transposase	Transposase, *Salinispora arenicola* (WP_028673963.1); 98.6/91	-
*kmy3*	375	Transposase	Transposase (or an inactivated derivative), *Micromonospora inyonensis* (SCL17972.1); 96.2/90	-
*kmy4*	921	Transcriptional regulator	LuxR family transcriptional regulator, *Candidatus Frankia datiscae* (WP_131764890.1); 38.9/99	-
*kmy5*	274	NAD(P)-dependent oxidoreductase	SPR family NAD(P)-dependent oxidoreductase, *Actinoplanes derwentensis* (WP_157751350.1); 71.2/100	-
*kmy6*	205	Transcriptional regulator	DNA-binding transcriptional regulator, AcrR family, *Actinoplanes derwentensis* (SDS69921.1); 67.3/98	-
*kmy7*	74	Hypothetical protein	Hypothetical protein, *Micromonospora sp.* M71_S20 (WP_121401669.1); 67.6/100	-
*kmy8*	149	Hypothetical protein	Hypothetical protein, *Streptomyces violaceoruber* (CAQ52628.1); 63.4/94	*ken18*
*kmy9*	517	FAD-dependent oxidoreductase	FAD-dependent oxidoreductase, *Streptomyces violaceoruber* (CAQ52629.1); 72.9/97	*ken19*
*kmy10*	3859	PKS I (module 4: KS, AT, DH, ER, KR, and ACP; module 5: KS, AT, DH, KR, and ACP)	Type I polyketide synthase, modules 4–5, *Streptomyces violaceoruber* (CAQ52622.1); 58.3/99	*ken12*
*kmy11*	2143	PKS I (module 6: KS, AT, DH, ER, KR, and ACP)	Type I polyketide synthase, modules 6, *Streptomyces violaceoruber* (CAQ52623.1); 60.4/99	*ken13*
*kmy12*	3346	PKS I (module 7: KS, AT, DH, ER, KR, and ACP; module 8: KS, AT, ACP, and TE)	Type I polyketide synthase, modules 7–8, *Streptomyces violaceoruber* (CAQ52624.1); 62.2/99	*ken14*
*kmy13*	390	FAD-dependent monooxygenase	FAD-dependent monooxygenase, *Streptomyces violaceoruber* (CAQ52625.1); 68.9/98	*ken15*
*kmy14*	486	Benzaldehyde dehydrogenase	Benzaldehyde dehydrogenase, *Streptomyces libani* (WP_190842308.1); 70.0/100	*ken6*
*kmy15*	551	Benzoylformate decarboxylase	Thiamine pyrophosphate-binding protein, *Streptomyces albus* subsp. *albus* (KUJ59733.1); 75.7/98	*ken5*
*kmy16*	444	Dioxygenase	Enoyl-CoA hydratase/isomerase family protein, Streptomyces griseus (WP_069170596.1); 66.7/90	*ken4*
*kmy17*	227	Enoyl-CoA hydratase/isomerase	Enoyl-CoA hydratase/isomerase family protein, *Streptomyces inusitatus* (WP_190127028.1); 56.6/92	*ken3*
*kmy18*	372	PKS III	Type III polyketide synthase, *Amycolatopsis anabasis* (WP_158891515.1); 72.8/95	*ken2*
*kmy19*	4494	PKS I (loading module: CAL, KR, and ACP; module 1: KS, AT, DH, KR, and ACP; module 2: KS, AT, DH, and KR)	Type I polyketide synthase, loading module and modules 1–3, *Streptomyces violaceoruber* (CAQ52626.1); 54.9/98	*ken16*
*kmy20*	1640	PKS I (module 3: ACP, KS, AT, KR, and ACP)	Type I polyketide synthase, loading module and modules 1–3, *Streptomyces violaceoruber* (CAQ52626.1); 58.8/99	*ken16*
*kmy21*	261	Type II Thioesterase	Thioesterase, *Streptacidiphilus melanoqenes* (WP 042389076.1); 56.3/93	-
*kmy22*	644	Methylmalonyl-CoA mutase	Methylmalonyl-CoA mutase small subunit, *Micromonospora krabiensis* (WP 091592824.1); 56.3/94	-
*kmy23*	723	Methylmalonyl-CoA mutase	Methylmalonyl-CoA mutase, *Micromonospora fluostatini* (TDB93065.1); 77.8/99	-
*kmy24*	343	Methylmalonyl-CoA mutase-associated GTPase	Methylmalonyl-CoA mutase-associated GTPase MeaB, *Nonomuraea rubra* (WP_185106477.1); 55.7/92	-
*kmy25*	433	Propionyl-CoA carboxylase	Acyl-CoA carboxylase subunit beta, *Amycolatopsis* sp. YIM10 (WP_153035332.1); 75.6/100	-
*kmy26*	547	PQQ-dependent enzyme	Polyvinyl alcohol dehydrogenase (cytochrome), *Allokutzneria albata* (SDN 68233.1); 69.5/90	*ken10*
*kmy27*	174	Hypothetical protein	Hypothetical protein, *Streptomyces abikoensis* (WP_18945037.1); 40.4/83	-
*kmy28*	125	DoxX family protein	DoxX family protein, *Streptomyces odonnellii* (WP 046501079.1); 45.6/100	-
*kmy29*	268	Enoyl-CoA hydratase/isomerase	Enoyl-CoA hydratase/isomerase family protein, *Phytohabitans houttuyneae* (WP_173055440.1); 78.7/100	*ken7*
*orf(+1)*	207	Hypothetical protein	Hypothetical protein, *Phytoactinopolyspora* sp. HAJB-30 (WP_166351888.1); 48.1/100	-
*orf(+2)*	67	Hypothetical protein	Hypothetical protein, *Phytoactinopolyspora* sp. HAJB-30 (WP_166351888.1); 62.5/95	-

^a^ Size in units of amino acids (aa); ID/SI: identity/similarity.

## Data Availability

The authors declare that all relevant data supporting the findings of this study are available within the article and its Supplementary Materials file, or from the corresponding authors upon request.

## Supplementary Materials

The following are available online at https://www.mdpi.com/article/10.3390/md19120673/s1, Figure S1: Structures of kendomycins, Figures S2–S10 and S19–S23: Gene disruption of *Verrucosispora* sp. SCSIO 07399, gene complementation of corresponding mutant strains and heterologous expression of *kmy* cluster, Figure S11: Chemical complementation of Δ*kmy18*, Figures S12–S18: Sequence alignments and phylogenetic analysis, Table S1: Strains and plasmids applied or constructed in this study, and Table S2: Primers used in this study.
