# Supplementary material for: Identification and Heterologous Expression of the Kendomycin B Biosynthetic Gene Cluster from Verrucosispora sp. SCSIO 07399
Source: Mar Drugs. 2021 Nov 26;19(12):673. doi: 10.3390/md19120673 (PMC8708025; doi:10.3390/md19120673)
Supplement: Supplementary file 1 [file marinedrugs-19-00673-s001.zip › marinedrugs-1465737-supplementary.pdf]

Supporting Information

Identification and heterologous expression of the kendomycin B biosynthetic gene cluster from *Verrucosispora* sp. SCSIO 07399

Jiang Chen [1,2,3,4], Shanwen Zhang [1,3], Yingying Chen [1,2,4], Xinpeng Tian [1], Yucheng Gu [5] and Jianhua Ju [1,2,3,4*]

1 CAS Key Laboratory of Tropical Marine Bio-resources and Ecology, Guangdong Key Laboratory of Marine Materia Medica, RNAM Center for Marine Microbiology, South China Sea Institute of Oceanology, Chinese Academy of Sciences, 164 West Xingang Road, Guangzhou 510301, China; chenjluck@126.com (J.C.); sherry920111@163.com (S.Z.); chenyingying7788@163.com (Y.C.); xinpengtian@scsio.ac.cn (X.T.)

2 Southern Marine Science and Engineering Guangdong Laboratory (Guangzhou), No.1119, Haibin Rd., Nansha District, Guangzhou 511458, China

3 College of Oceanology, University of Chinese Academy of Sciences, Qingdao 266400, China

4 Sanya Institute of Oceanology, SCSIO, Zhenzhou Road, Yazhou District, Sanya 572000, China

5 Syngenta Jealott's Hill International Research Centre, Bracknell, Berkshire RG42 6EY, UK; yucheng.gu@syngenta.com (Y.G.)

* Correspondence: jju@scsio.ac.cn (J.J.); Tel./Fax: +86-20-8902-3028 (J.J.)

Item Name	Contents/Description	Page
**Table S1.**	Strains and plasmids applied or constructed in this study.	S2
**Table S2.**	Primers used in this study.	S3-S4
**Figure S1.**	Structures of kendomycins.	S5
**Figure S2.**	Construction of *kmy11* gene mutant strain of *Verrucosispora* sp. SCSIO 07399 by PCR-targeting technique.	S5
**Figure S3.**	Construction of *kmy18* gene mutant strain of *Verrucosispora* sp. SCSIO 07399 by PCR-targeting technique.	S5
**Figure S4.**	HPLC analysis of fermentation broths from gene mutant strains.	S6
**Figure S5.**	Construction of *orf(-2)-kmy3* genes mutant strain of *Verrucosispora* sp. SCSIO 07399 by PCR-targeting technique.	S6
**Figure S6.**	Construction of *kmy27-orf(+2)* genes mutant strain of *Verrucosispora* sp. SCSIO 07399 by PCR-targeting technique.	S7
**Figure S7.**	Construction of *kmy29* gene mutant strain of *Verrucosispora* sp. SCSIO 07399 by PCR-targeting technique.	S7
**Figure S8.**	Construction of *kmy29* gene complemented strain by site-specific recombinase φC31 system.	S8
**Figure S9.**	Construction of *kmy22-25* genes mutant strain of *Verrucosispora* sp. SCSIO 07399 by PCR-targeting technique.	S8
**Figure S10.**	Construction of *kmy26* gene mutant strain of *Verrucosispora* sp. SCSIO 07399 by PCR-targeting technique.	S9
**Figure S11.**	Chemical complementation of type III PKS gene mutant strain Δ*kmy18*.	S9
**Figure S12.**	Sequence alignment of type I PKS domains from kendomycin B gene cluster.	S10
**Figure S13.**	Sequence alignment of TE domains.	S10
**Figure S14.**	Phylogenetic analysis of Kmy12_TE and other thioesterases.	S11
**Figure S15.**	Sequence analysis of Kmy9 and Kmy13.	S11
**Figure S16.**	Phylogenetic analysis of Kmy13.	S12
**Figure S17.**	Phylogenetic analysis of Kmy4 and other LuxR-type regulators.	S12
**Figure S18.**	Sequence alignment of Kmy4 with several representative LAL-type of LuxR family regulators.	S13
**Figure S19.**	Construction of *kmy4* gene mutant strain of *Verrucosispora* sp. SCSIO 07399 by PCR-targeting technique.	S13
**Figure S20.**	Construction of *kmy4* gene complemented strain by site-specific recombinase φC31 system.	S14
**Figure S21.**	Construction of *kmy6* gene mutant strain of *Verrucosispora* sp. SCSIO 07399 by PCR-targeting technique.	S14
**Figure S22.**	HPLC analysis of fermentation broths from regulatory gene mutants.	S15
**Figure S23.**	PCR identification of the genotype of *kmy* gene cluster hetero-expression strain.	S15
**-**	Supplemental references.	S16

Table S1. Strains and plasmids applied or constructed in this study.

Strains or plasmids	Description	Reference or source
**Strains**		
E. coli LE392	Host strain of cosmid vector SuperCosI	Stratagene
E. coli DH10β	Host strain of plasmid vector pHZAUFXJ	Stratagene
E. coli BW25113/pIJ790	Strain for homologous reorganization during PCR-targeting process	1
E. coli DH5α/pIJ773	Host strain of plasmid pIJ773	Stratagene
E. coli ET12567/pUZ8002	Strain for conjugation with Streptomyces	2
E. coli ET12567/pUB307	Strain for triparental conjugation with Streptomyces	3
E. coli DH5α/pL646ATE-ssaA-BXSE	Host strain of plasmid pL646ATE-ssaA-BXSE	Our lab
Verrucosispora sp. SCSIO 07399	Producing strain of kendomycin B-D	This study
Streptomyces coelicolor M1152	Heterologous expression host	3
S. coelicolor M1152::pHZAUFXJ-3-J11	Kendomycin gene cluster hetero-expression strain	This study
Verrucosispora sp. SCSIO 07399/Δ*orf(-2)-kmy3*	*orf(-2)-kmy3* genes mutant strain derived from *Verrucosispora* sp. SCSIO 07399	This study
Verrucosispora sp. SCSIO 07399/Δ*kmy22-25*	*kmy22-25* genes mutant strain derived from *Verrucosispora* sp. SCSIO 07399	This study
Verrucosispora sp. SCSIO 07399/Δ*kmy27-orf(+2)*	*kmy27-orf(+2)* genes mutant strain derived from *Verrucosispora* sp. SCSIO 07399	This study
Verrucosispora sp. SCSIO 07399/Δ*kmy4*	*kmy4* gene mutant strain derived from *Verrucosispora* sp. SCSIO 07399	This study
Verrucosispora sp. SCSIO 07399/Δ*kmy6*	*kmy6* gene mutant strain derived from *Verrucosispora* sp. SCSIO 07399	This study
Verrucosispora sp. SCSIO 07399/Δ*kmy11*	*kmy11* gene mutant strain derived from *Verrucosispora* sp. SCSIO 07399	This study
Verrucosispora sp. SCSIO 07399/Δ*kmy18*	*kmy18* gene mutant strain derived from *Verrucosispora* sp. SCSIO 07399	This study
Verrucosispora sp. SCSIO 07399/Δ*kmy26*	*kmy26* gene mutant strain derived from *Verrucosispora* sp. SCSIO 07399	This study
Verrucosispora sp. SCSIO 07399/Δ*kmy29*	*kmy29* gene mutant strain derived from *Verrucosispora* sp. SCSIO 07399	This study
Verrucosispora sp. SCSIO 07399/Δ*kmy4*::*kmy4*	*kmy4* gene complemented strain derived from *Verrucosispora* sp. SCSIO 07399/Δ*kmy4*	This study
Verrucosispora sp. SCSIO 07399/Δ*kmy27-orf(+2)*::*kmy29*	*kmy29* gene complemented strain derived from *Verrucosispora* sp. SCSIO 07399/Δ*kmy27-orf(+2)*	This study
**Plasmids**		
SuperCosI	Amp^r, Kan^r, cosmid vector	Stratagene
pHZAUFXJ	Apr^r, plasmid vector	Stratagene
pIJ790	Cml^r; *gam, bet, exo* encoding λ-RED reorganization system for PCR-targeting	4
pIJ773	Apr^r, provider of *oriT/acc(3)IV* cassette	4
pUZ8002	Kan^r, non-transmissible plasmid with *tra* gene for conjugation	5
pUB307	Kan^r, transmissible plasmid with *tra* gene for conjugation	3
pL646ATE-ssaA-BXSE	Apr^r, Tsr^r, ermE*p1&p2, complemented vector	Derived from pL646

Table S2. Primers used in this study.

Primers	Sequence (5′ - 3′)
**For genomic library screening**	
kmy3-SF	GCGAAGACGGTGCAGACCGA
kmy3-SR	CCGGACGAGGTACACGACGC
kmy13-SF	GTCTGGACGGACATGCGGGA
kmy13-SR	GGCTCACGTACGTCACGCAG
kmy28-SF	GAACTGGTAATCGCCGAAGCG
kmy28-SR	GCAAAGTTCGTCGTCCCGATC
orf(+4)-SF	CTCTGGCATGGAAGCGTCG
orf(+4)-SR	CGCGCATAGTGTGGGTTATG
**For construction of gene mutant strain**	
orf(-2)-kmy3-delF	GCGCACGGCATCGACAGCAACTACGCCAACTGTATCGTGattccggggatccgtcgacc
orf(-2)-kmy3-delR	GGTGGTGTAGAGGACCTTGCGGACCTCGTGGTCGTAGTCtgtaggctggagctgcttc
orf(-2)-kmy3-testF	CGACTCCATCGACATCCGCC
orf(-2)-kmy3-testR	GGACCTTCATCGCGGCCTG
kmy22-25-delF	GTGATGATGACCGTCCGGGATTCGTGGACCAACGCGCTGattccggggatccgtcgacc
kmy22-25-delR	GATCACCCTGGTCGCGCCGAGCGACTTGGAGTTCATCGCtgtaggctggagctgcttc
kmy22-25-testF	CACCGGCACCACCATCGC
kmy22-25-testR	GCTCGGCGATCAGCCGTG
kmy27-orf(+2)-delF	GTTGTACGACAGGCTCACGGCCACACTGGTGCCGTTCGCattccggggatccgtcgacc
kmy27-orf(+2)-delR	GCCGAGGATCGCGTCACCCACATGGATCAGCGAGCACACtgtaggctggagctgcttc
kmy27-orf(+2)-testF	GACGGAAGAGGTAGGCCCCG
kmy27-orf(+2)-testR	CGAGCGCCGAAGTCGCTG
kmy4-delF	GGAGTCGACGACGCCGAGTACGCGGACACGGCCTCGCTGattccggggatccgtcgacc
kmy4-delR	CCGGCCGGCCCGGAGCAGGTCCACGAGCTGAGGAAGGTGtgtaggctggagctgcttc
kmy4-testF	CTGATCGCGGAGGCTCGCC
kmy4-testR	GCCGCCCGTACCAGAGCAG
kmy6-delF	GAGGCCTTCGCCGAGCACGGTCTGGCGCTCGACGTACGGattccggggatccgtcgacc
kmy6-delR	GACCGGCTGGAGTTCGTCCTGGCACTGCTGGAAGCGCGGtgtaggctggagctgcttc
kmy6-testF	GACGCCGTCGACAACCGC
kmy6-testR	CGCAGCACACCGGCGTC
kmy11-delF	GTCATGTACCACGACTACGCCGCCCGGCTGACCGAGATCattccggggatccgtcgacc
kmy11-delR	GCGTACCAAGGCAAGGATCTTGTGGTTGTTGCGGCGTGCtgtaggctggagctgcttc
kmy11-testF	CCTGGGAGACGTTCGAGCG
kmy11-testR	GATCACCCGCTGCTGCGAAG
kmy18-delF	GACGCCGACCTGTCCGATGTGCGATACCTCTGCTGCGTCattccggggatccgtcgacc
kmy18-delR	GGAATCCCAGTCGTAACGCATTGCGTCGACTGCCTCGGGtgtaggctggagctgcttc
kmy18-testF	GAGAGTCAGGCCGACCTGC
kmy18-testR	CCGATGATCGTTTCCGCGTG
kmy26-delF	GGCAAGGTCTACTTCGGCGACTACCGGGGCTACCTCTACattccggggatccgtcgacc
kmy26-delR	CACGATCTCGCCGGTCCGTGCGTCGAAGGTGTGGTAGACtgtaggctggagctgcttc
kmy26-testF	CTTCTACGCCGTGGACGCC
kmy26-testR	GTTCAGCGCGAAGAGCGTG
kmy29-delF	GAGGTACTGAACGCCATGGACACGCACATGCACGCCGAGattccggggatccgtcgacc
kmy29-delR	CAGATCGTCCACCCAGCCCGCCACGCACTCGTCCAGTTGtgtaggctggagctgcttc
kmy29-testF	GGTGCGGTACGCGAAACGC
kmy29-testR	GCATGTCGACGGACCGCAG
**For construction of gene complemented strain**	
kmy4-cplF	GCTTCATATGATGGTGGACAGCGACAGG
kmy4-cplR	AATTGGATCCTCATGCCGTTTCGGCCAG
kmy29-cplF	GGCATATGATGGCAGAGGACGACAGGCC

kmy29-cplR	GGATCCTCAGCGGCCCTCCCAGC
**For real-time qPCR**	
RT-*kmy9*-F	CGTCATCATCATGAGCATCC
RT-*kmy9*-R	GCAGATAGGCGGTCTTGAAC
RT-*kmy10*-F	CGTATTACGCGTTGGTGGAT
RT-*kmy10*-R	ACACCCACCACACTCCACTT
RT-*kmy11*-F	GACGCGGACTACTGGTATCG
RT-*kmy11*-R	AGATACAGCCGTTGCAGACC
RT-*kmy13*-F	CACCAGGTGGAAGTGTACGA
RT-*kmy13*-R	GTCCGTCCAGACCAGCAG
RT-*kmy16*-F	AGGTCGAGGACCTGGAGACA
RT-*kmy16*-R	TCTGGATGTAGCCGATCTCC
RT-*kmy18*-F	ATGCGTTACGACTGGGATTC
RT-*kmy18*-R	CGAGTCGATGACCTTCTTCC
RT-*kmy19*-F	CCTGTTCCTCCTCACTGGTC
RT-*kmy19*-R	GGCGCTCCAGCAGTAGTAGT
RT-*kmy23*-F	GACCATCCAGAACGACATCC
RT-*kmy23*-R	GTGGTATCCGGAGATCGAGA
RT-*16S*-F	CTCTTCGACCTCGGTGTCTC
RT-*16S*-R	GAACTTCTCCTCGCCGTACA

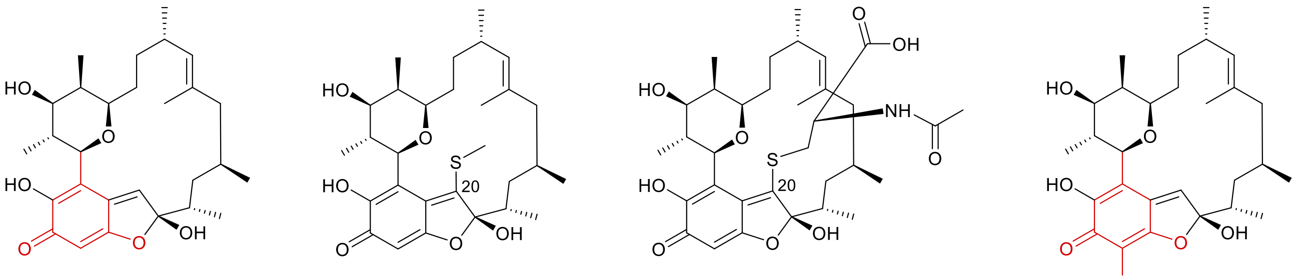

kendomycin B (**1**) kendomycin C (**2**) kendomycin D (**3**) kendomycin

Figure S1. Structures of kendomycins. Kendomycin B-D (**1-3**) purified from *Verrucosispora* sp. SCSIO 07399 [6] and kendomycin produced by *Streptomyces violaceoruber* strain 3844-33C reported previously.

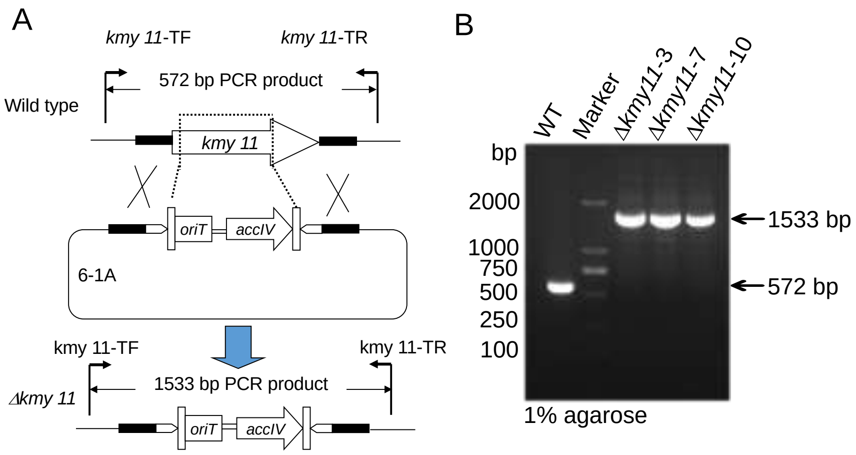

Figure S2. Construction of *kmy11* gene mutant strain of *Verrucosispora* sp. SCSIO 07399 by PCR-targeting technique. (A) Schematic diagram of gene *kmy11* disruption. (B) Verification of the gene *kmy11* double-cross mutants by comparing with the wild-type strain *via* PCR using the primers listed in **Table S2**. WT: wild-type strain; Δ*kmy11*: gene *kmy11* mutant strains. Marker: DL2000 DNA Marker (Takara).

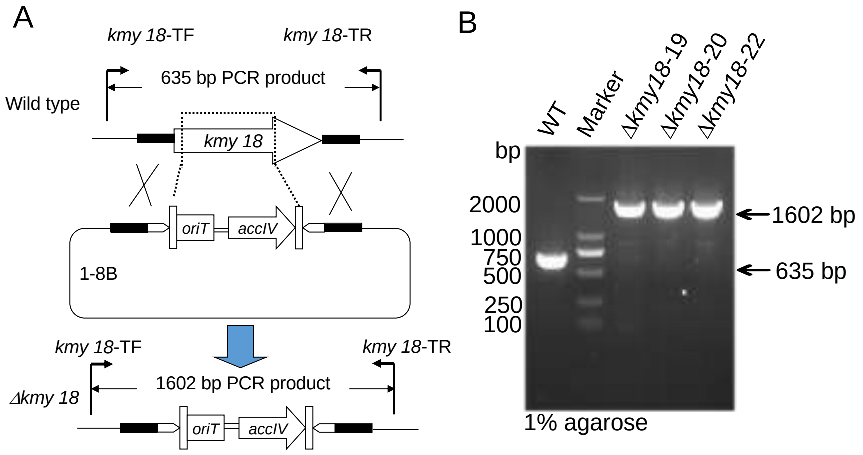

Figure S3. Construction of *kmy18* gene mutant strain of *Verrucosispora* sp. SCSIO 07399 by PCR-targeting technique. (A) Schematic diagram of gene *kmy18* disruption. (B) Verification of the gene *kmy18* double-cross mutants by comparing with the wild-type strain *via* PCR using the primers listed in **Table S2**. WT: wild-type strain; Δ*kmy18*: gene *kmy18* mutant strains. Marker: DL2000 DNA Marker (Takara).

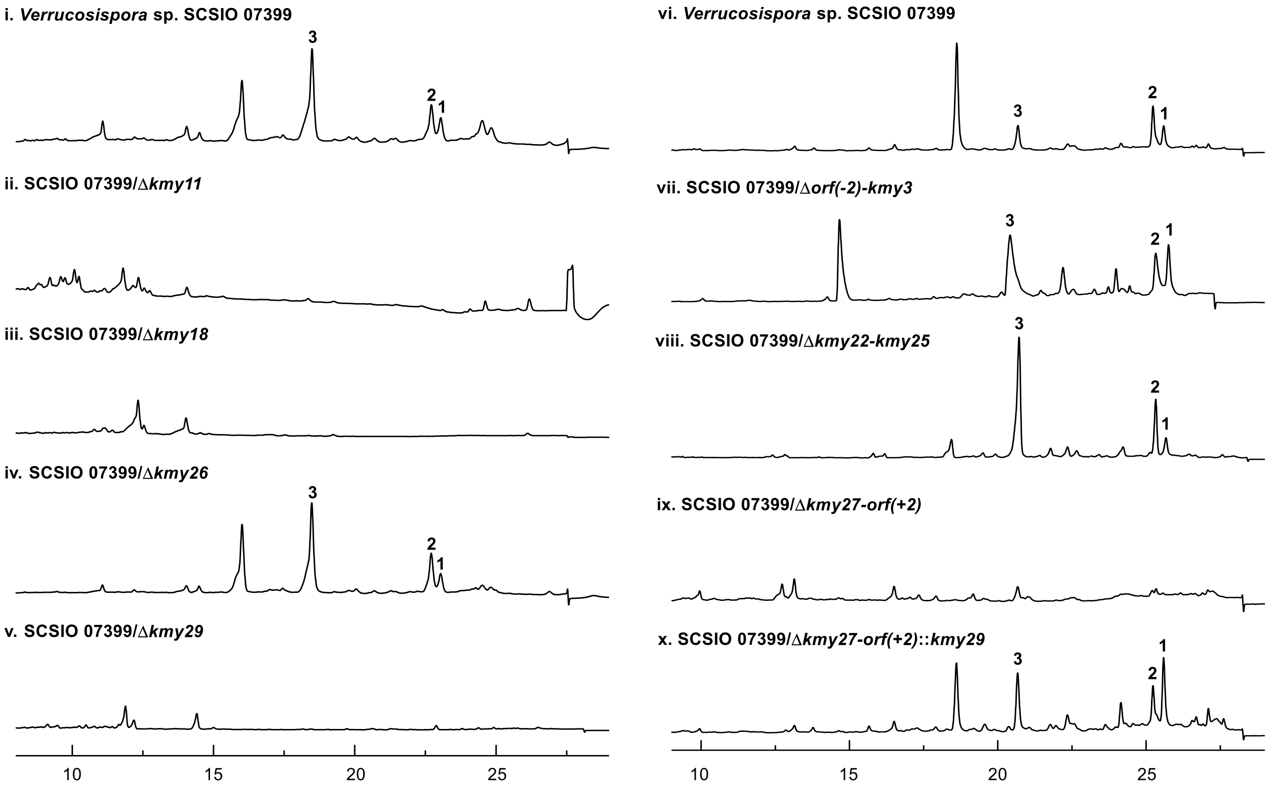

Figure S4. HPLC analysis (UV at 285 nm) of fermentation broths from (i) the wild-type strain *Verrucosispora* sp. SCSIO 07399; (ii) mutant strain *Verrucosispora* sp. SCSIO 07399/Δ*kmy11*; (iii) mutant strain *Verrucosispora* sp. SCSIO 07399/Δ*kmy18*; (iv) mutant strain *Verrucosispora* sp. SCSIO 07399/Δ*kmy26*; (v) mutant strain *Verrucosispora* sp. SCSIO 07399/Δ*kmy29*; (vi) the wild-type strain; (vii) mutant strain *Verrucosispora* sp. SCSIO 07399/Δ*orf(-2)-kmy3*; (viii) mutant strain *Verrucosispora* sp. SCSIO 07399/Δ*kmy22-25*; (ix) mutant strain *Verrucosispora* sp. SCSIO 07399/Δ*kmy27-orf(+2)*; (x) complemented strain *Verrucosispora* sp. SCSIO 07399/Δ*kmy27-orf(+2)*::*kmy29*.

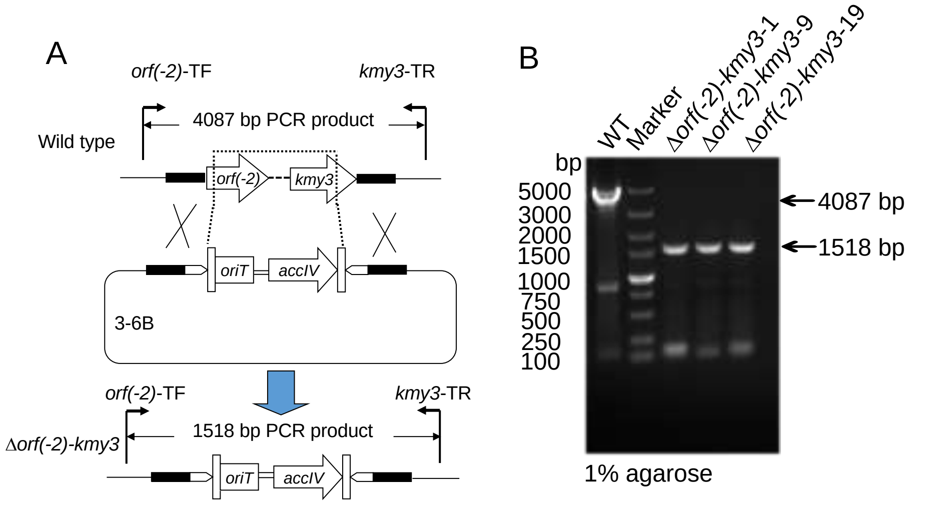

Figure S5. Construction of *orf(-2)-kmy3* genes mutant strain of *Verrucosispora* sp. SCSIO 07399 by PCR-targeting technique. (A) Schematic diagram of genes *orf(-2)-kmy3* disruption. (B) Verification of the genes *orf(-2)-kmy3* double-cross mutants by comparing with the wild-type strain *via* PCR using the primers listed in **Table S2**. WT: wild-type strain; Δ*orf(-2)-kmy3*: genes *orf(-2)-kmy3* mutant strains. Marker: DL5000 DNA Marker (Takara).

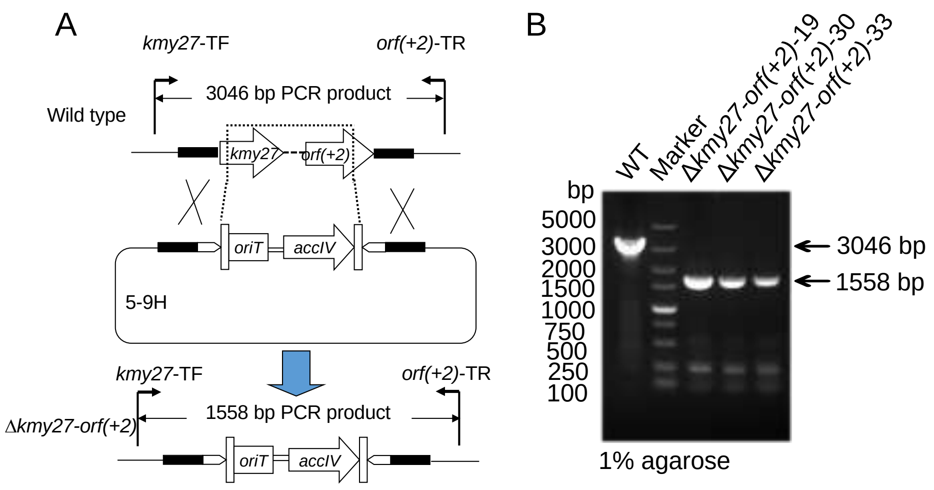

Figure S6. Construction of *kmy27-orf(+2)* genes mutant strain of *Verrucosispora* sp. SCSIO 07399 by PCR-targeting technique. (A) Schematic diagram of genes *kmy27-orf(+2)* disruption. (B) Verification of the genes *kmy27-orf(+2)* double-cross mutants by comparing with the wild-type strain *via* PCR using the primers listed in **Table S2**. WT: wild-type strain; Δ*kmy27-orf(+2)*: genes *kmy27-orf(+2)* mutant strains. Marker: DL5000 DNA Marker (Takara).

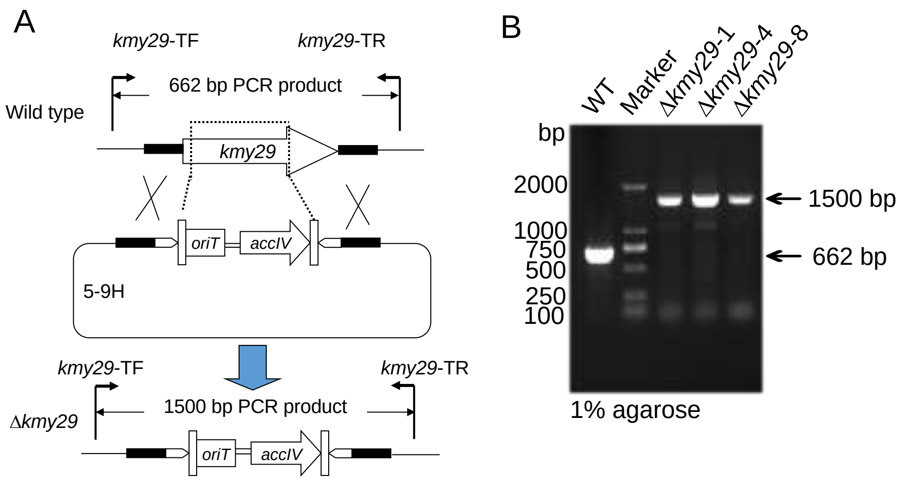

Figure S7. Construction of *kmy29* gene mutant strain of *Verrucosispora* sp. SCSIO 07399 by PCR-targeting technique. (A) Schematic diagram of gene *kmy29* disruption. (B) Verification of the gene *kmy29* double-cross mutants by comparing with the wild-type strain *via* PCR using the primers listed in **Table S2**. WT: wild-type strain; Δ*kmy29*: gene *kmy29* mutant strains. Marker: DL2000 DNA Marker (Takara).

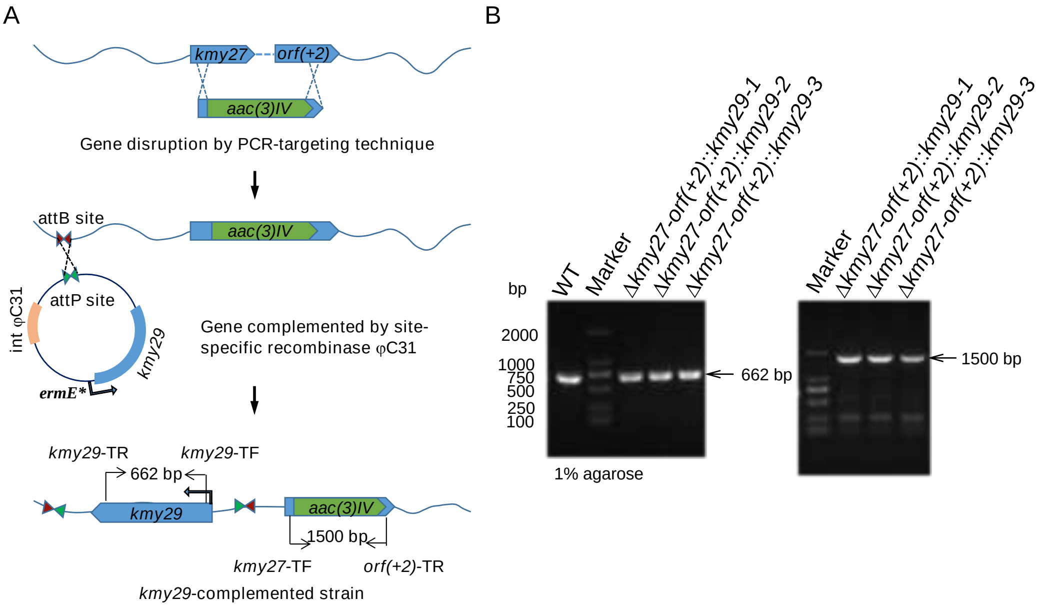

Figure S8. Construction of *kmy29* gene complemented strain of *Verrucosispora* sp. SCSIO 07399/Δ*kmy27-orf(+2)* by site-specific recombinase φC31 system. (A) Schematic diagram of gene complement process. (B) Verification of the gene *kmy29* complemented strains by comparing with the wild-type strain *via* PCR using the primers listed in **Table S2**. WT: wild-type strain; Δ*kmy27-orf(+2)*::*kmy29*: gene *kmy29* complemented strains. Marker: DL2000 DNA Marker (Takara).

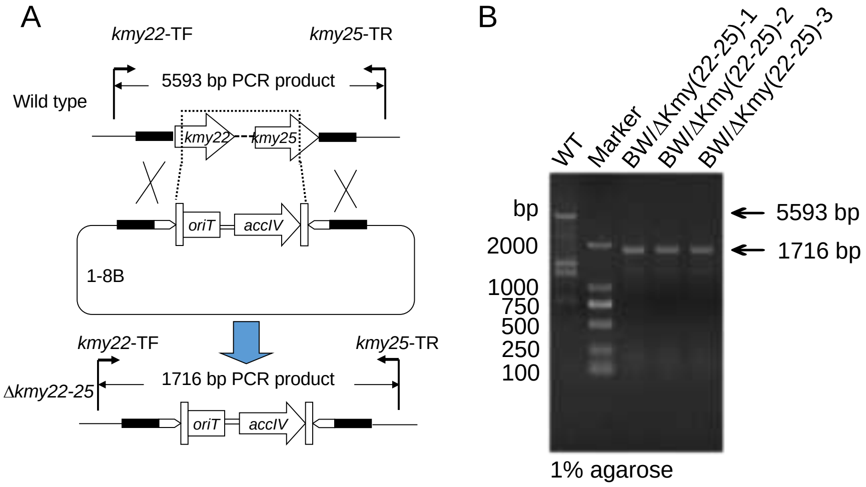

Figure S9. Construction of *kmy22-25* genes mutant strain of *Verrucosispora* sp. SCSIO 07399 by PCR-targeting technique. (A) Schematic diagram of genes *kmy22-25* disruption. (B) Verification of the genes *kmy22-25* double-cross mutants by comparing with the wild-type strain *via* PCR using the primers listed in **Table S2**. WT: wild-type strain; Δ*kmy22-25*: genes *kmy22-25* mutant strains. Marker: DL2000 DNA Marker (Takara).

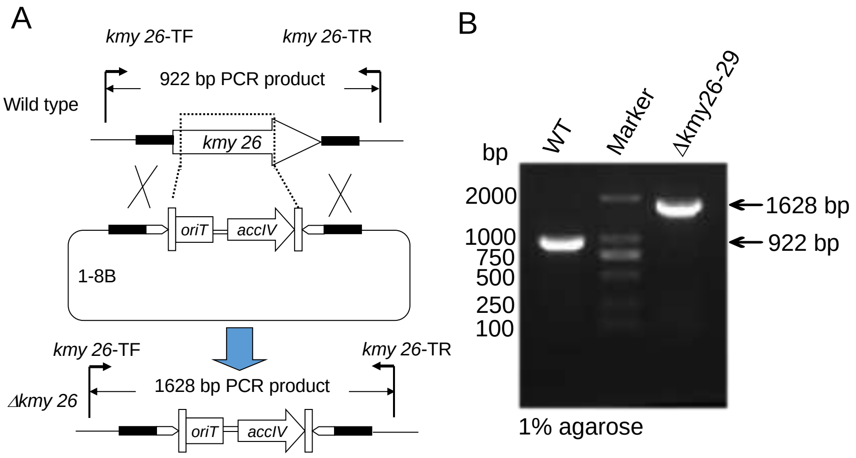

Figure S10. Construction of *kmy26* gene mutant strain of *Verrucosispora* sp. SCSIO 07399 by PCR-targeting technique. (A) Schematic diagram of gene *kmy26* disruption. (B) Verification of the gene *kmy26* double-cross mutants by comparing with the wild-type strain *via* PCR using the primers listed in **Table S2**. WT: wild-type strain; Δ*kmy26*: gene *kmy26* mutant strains. Marker: DL2000 DNA Marker (Takara).

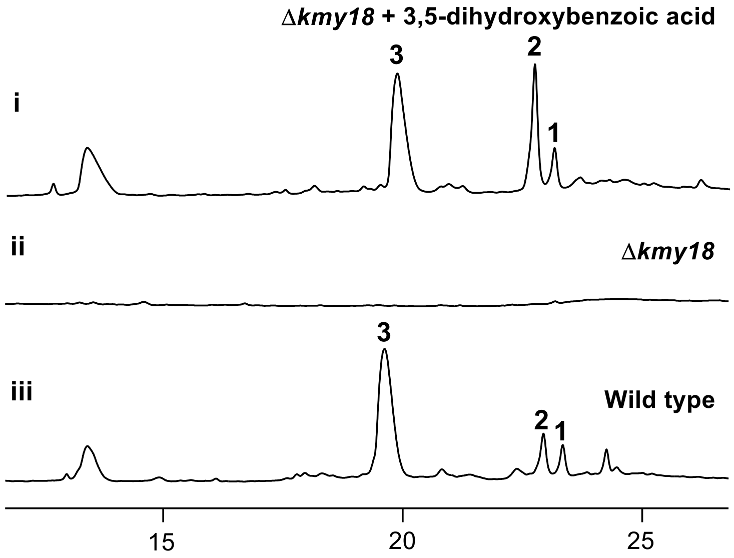

Figure S11. Chemical complementation of type III PKS gene mutant strain Δ*kmy18*. HPLC analysis (UV at 285 nm) of fermentation broths from (i) Δ*kmy18* feeding with 3,5-dihydroxybenzoic acid; (ii) mutant strain Δ*kmy18*; (iii) the wild-type strain *Verrucosispora* sp. SCSIO 07399.

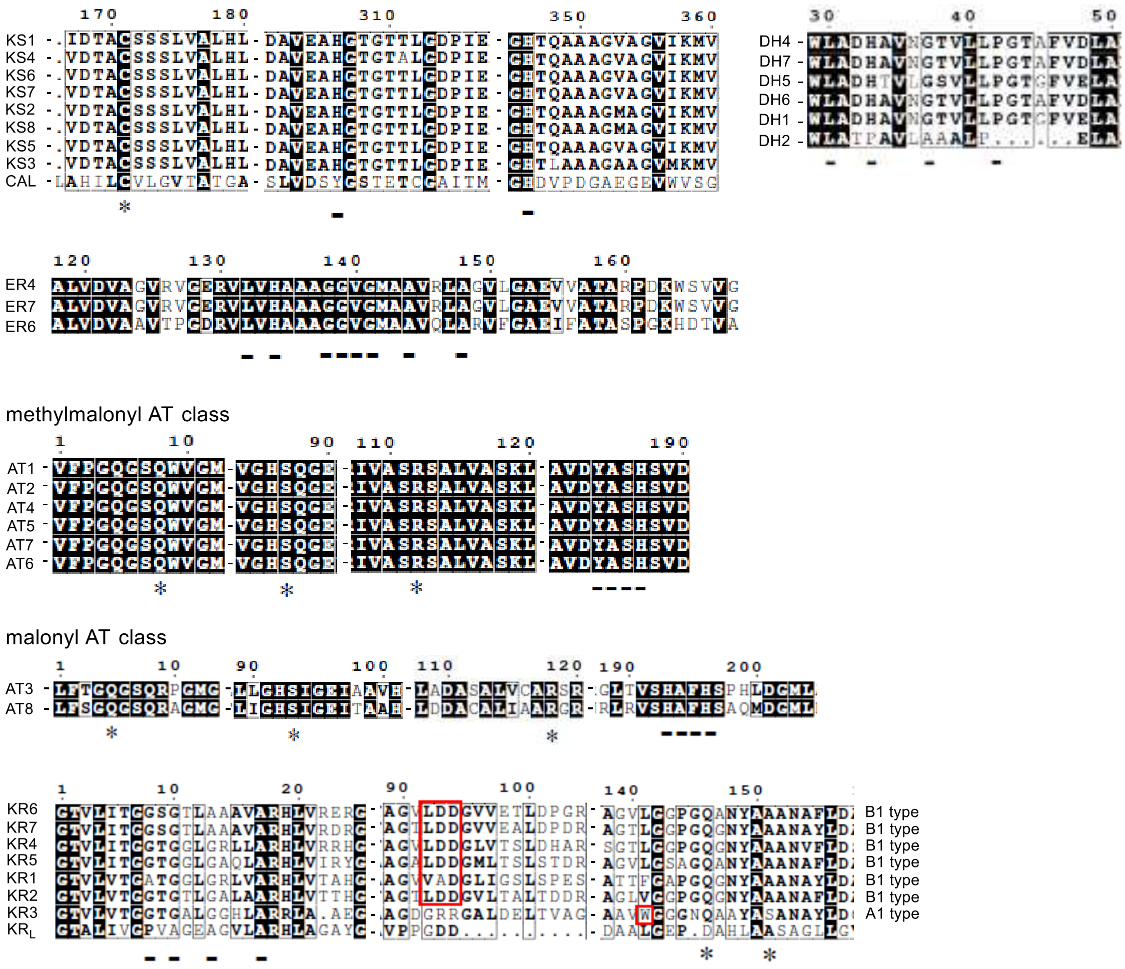

Figure S12. Sequence alignment of type I PKS domains from kendomycin B gene cluster, but only the key regions were shown. Key residues were marked with asterisks and conserved motifs were underlined. The characteristic motifs of A-type and B-type KR (ketoreductase) domains had been framed by red solid lines.

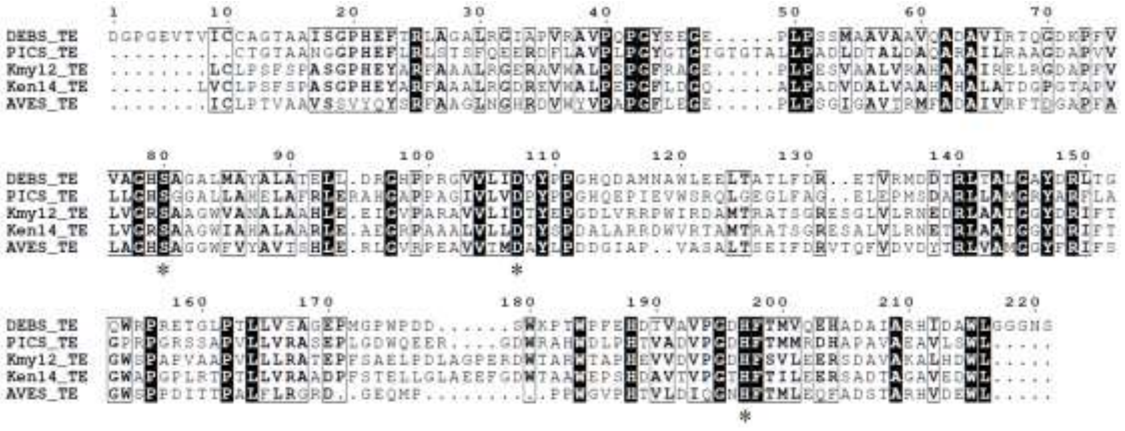

Figure S13. Sequence alignment of Kmy12_TE with Ken14_TE (CAQ52624.1) from kendomycin biosynthetic gene cluster (BGC) and macrocycle-forming TEs: DEBS_TE from 6-deoxyerythronolide BGC (X62569.1); PICS_TE from picromycin BGC (AF079138.1); AVES_TE from avermectin BGC (BAA84479.1). The characteristic Ser (S), Asp (D), His (H) catalytic triad was marked by asterisks.

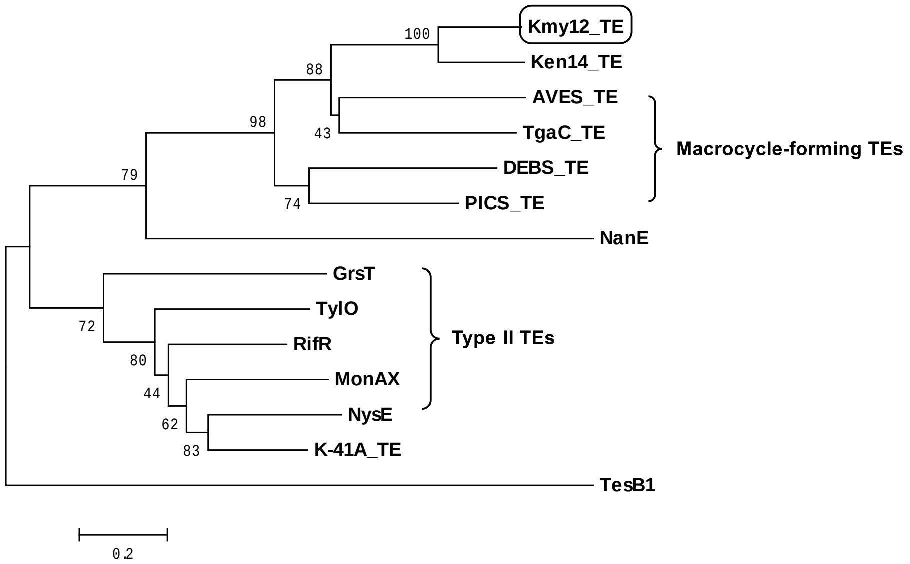

Figure S14. Phylogenetic analysis of Kmy12_TE and other thioesterases.

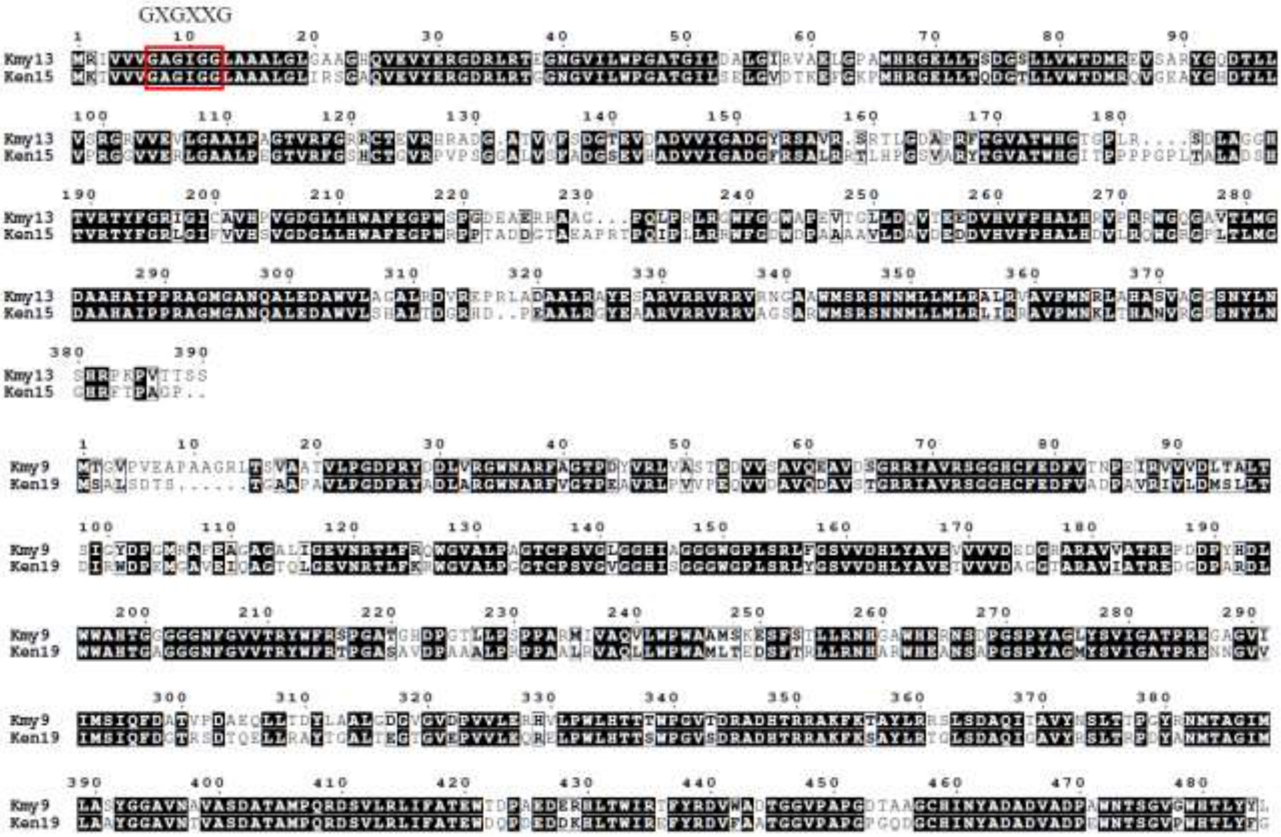

Figure S15. Sequence analysis of FAD-dependent oxidoreductase Kmy9 and FAD-dependent monooxygenase Kmy13 by comparing with their corresponding homologues. The characteristic GxGxxG fingerprint was highlighted.

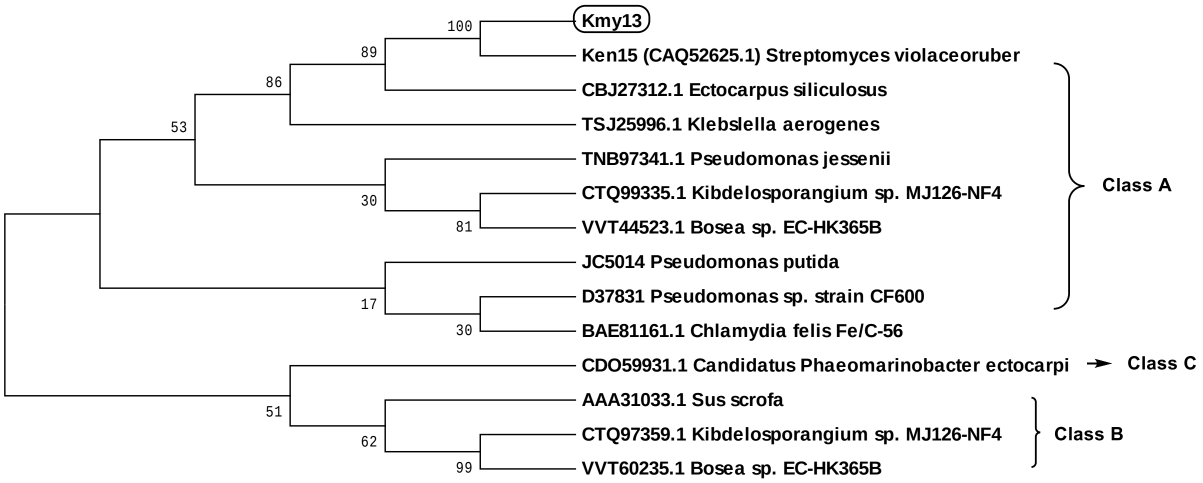

Figure S16. Phylogenetic analysis of Kmy13. Class D-F flavoprotein monooxygenases were all encoded by two genes encoding a monooxygenase and a reductase, so we didn't use them to construct the phylogenic tree.

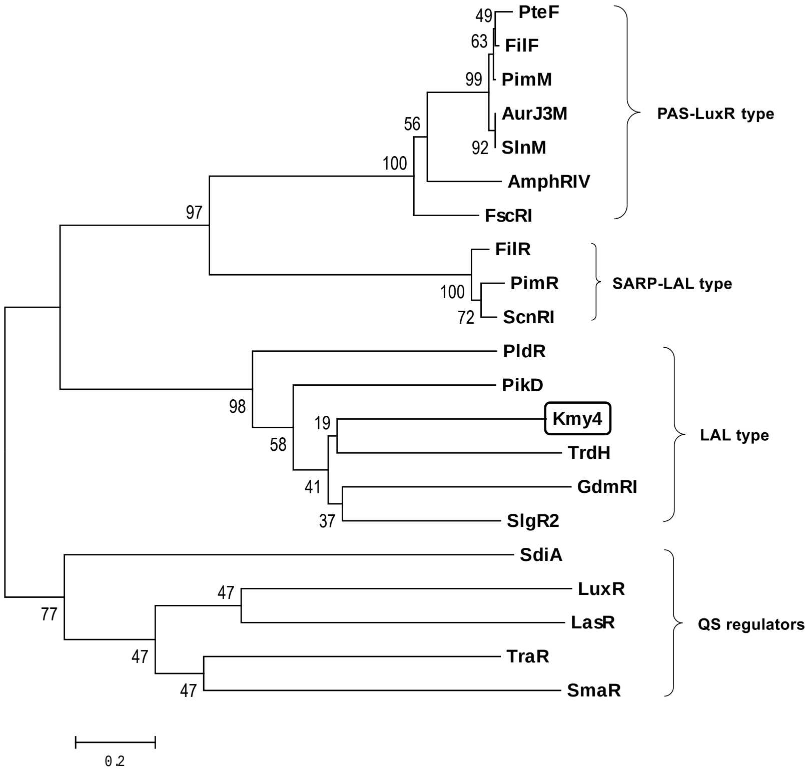

Figure S17. Phylogenetic analysis of Kmy4 and other LuxR-type regulators.

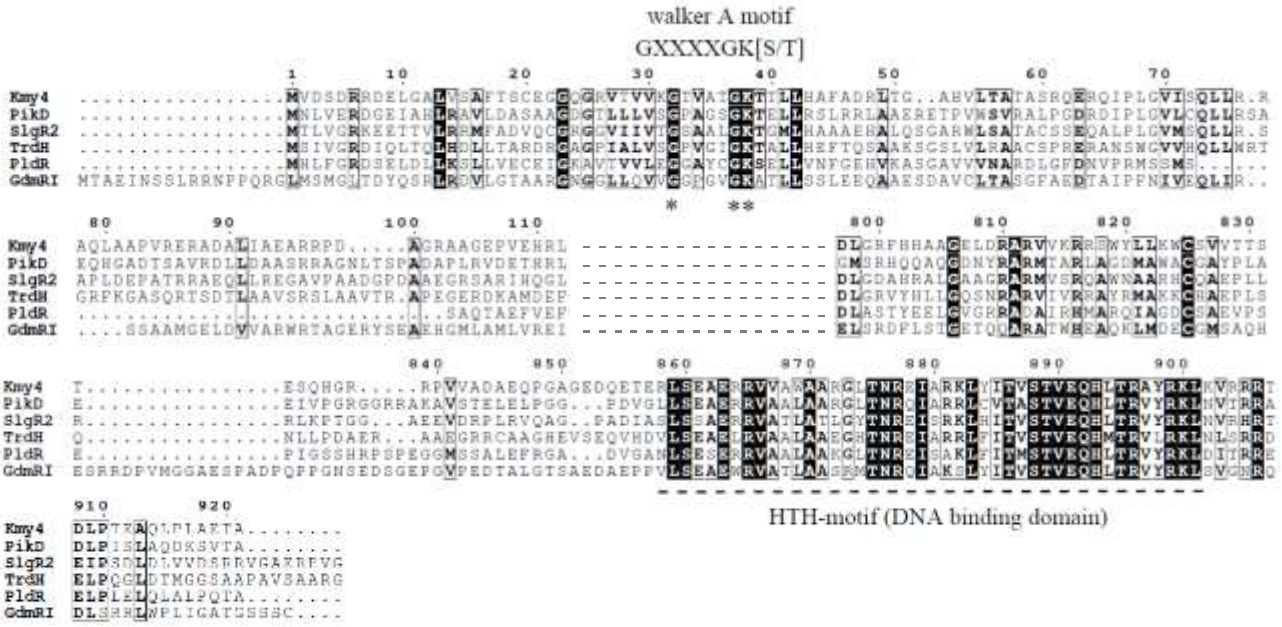

Figure S18. Sequence alignment of Kmy4 with several representative LAL-type LuxR family regulators (just presented the key regions). The conserved walker A motif GXXXXGK[S/T] was marked by asterisks, and the putative helix-turn-helix (HTH)-DNA binding motif was underlined by dotted line.

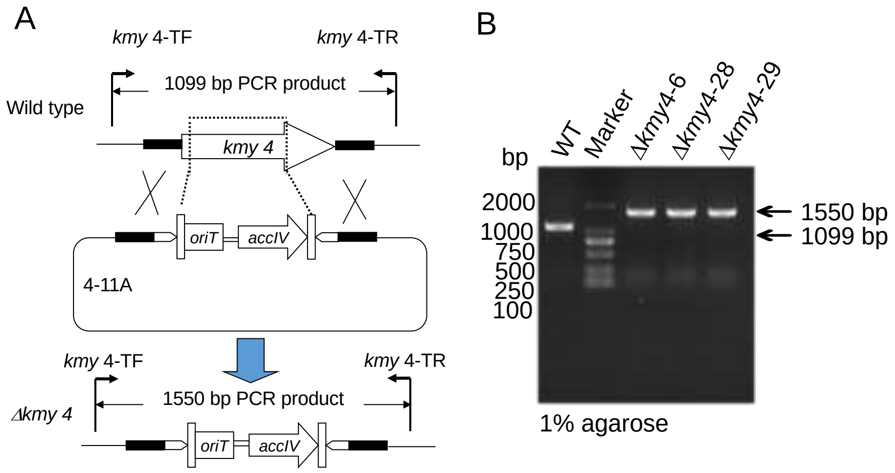

Figure S19. Construction of *kmy4* gene mutant strain of *Verrucosispora* sp. SCSIO 07399 by PCR-targeting technique. (A) Schematic diagram of gene *kmy4* disruption. (B) Verification of the gene *kmy4* double-cross mutants by comparing with the wild-type strain *via* PCR using the primers listed in **Table S2**. WT: wild-type strain; Δ*kmy4*: gene *kmy4* mutant strains. Marker: DL2000 DNA Marker (Takara).

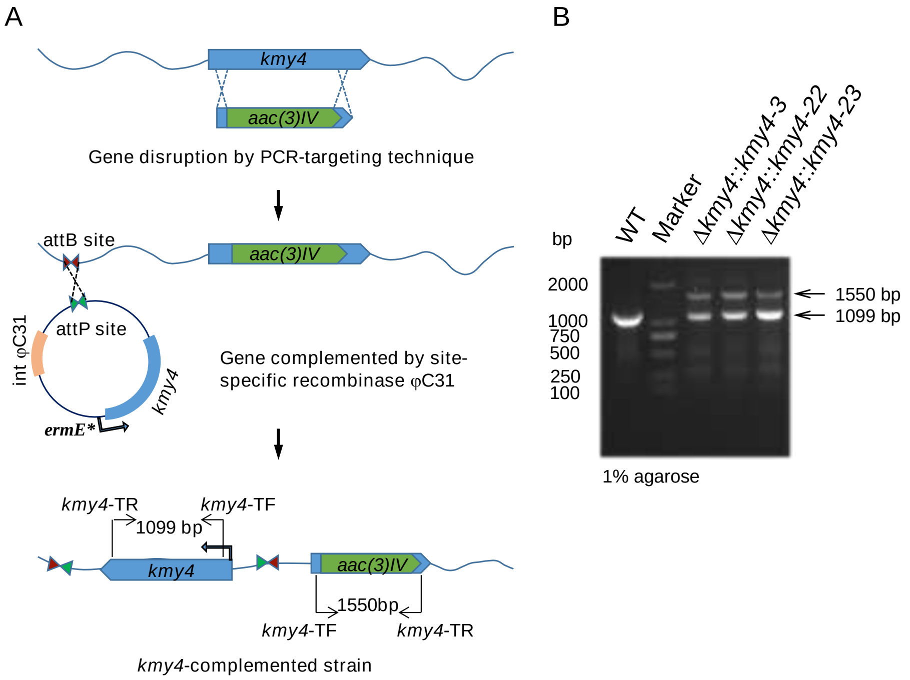

Figure S20. Construction of *kmy4* gene complemented strain of *Verrucosispora* sp. SCSIO 07399/Δ*kmy4* by site-specific recombinase φC31 system. (A) Schematic diagram of gene complement process. (B) Verification of the gene *kmy4* complemented strains by comparing with the wild-type strain *via* PCR using the primers listed in **Table S2**. WT: wild-type strain; Δ*kmy4*::*kmy4*: gene *kmy4* complemented strains. Marker: DL2000 DNA Marker (Takara).

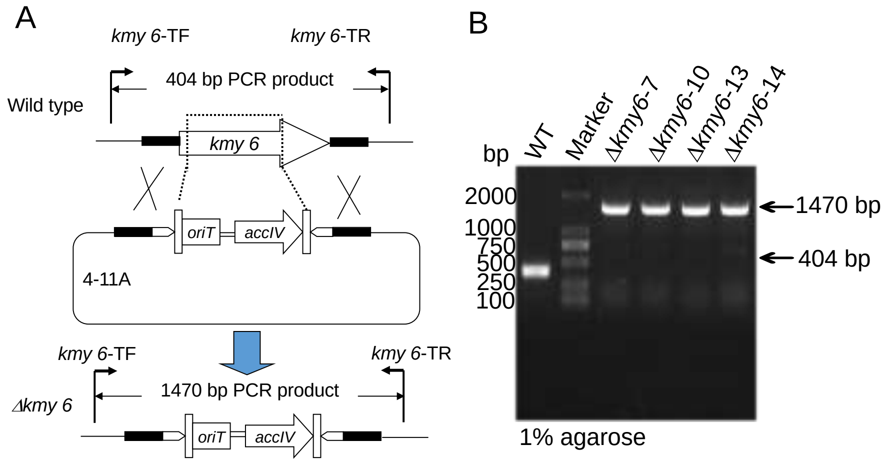

Figure S21. Construction of *kmy6* gene mutant strain of *Verrucosispora* sp. SCSIO 07399 by PCR-targeting technique. (A) Schematic diagram of gene *kmy6* disruption. (B) Verification of the gene *kmy6* double-cross mutants by comparing with the wild-type strain *via* PCR using the primers listed in **Table S2**. WT: wild-type strain; Δ*kmy6*: gene *kmy6* mutant strains. Marker: DL2000 DNA Marker (Takara).

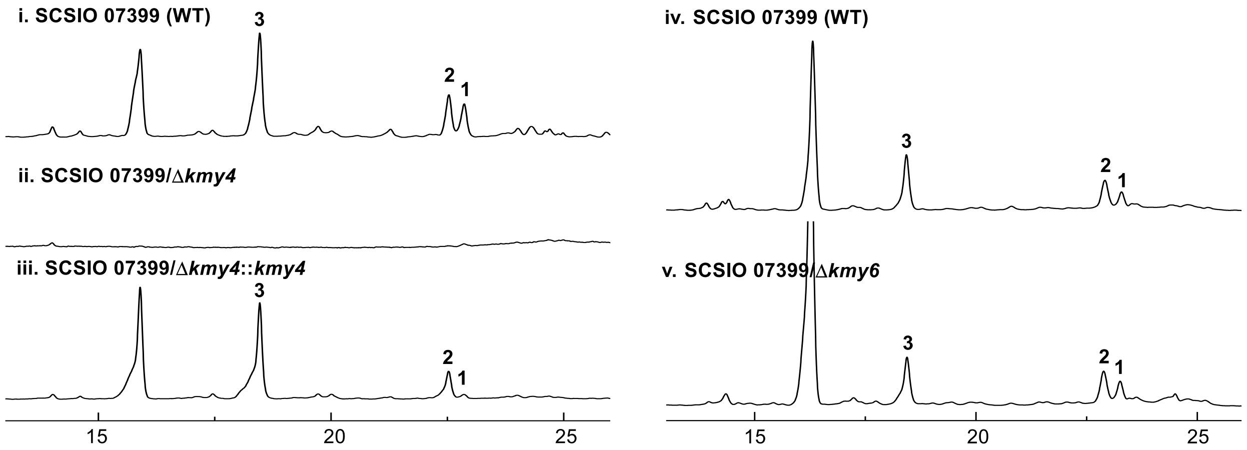

Figure S22. HPLC analysis (UV at 285 nm) of fermentation broths from regulatory gene mutants. (i) the wild-type strain *Verrucosispora* sp. SCSIO 07399; (ii) mutant strain *Verrucosispora* sp. SCSIO 07399/Δ*kmy4*; (iii) *kmy4* gene complemented strain *Verrucosispora* sp. SCSIO 07399/Δ*kmy4*::*kmy4*; (iv) the wild-type strain; (v) mutant strain *Verrucosispora* sp. SCSIO 07399/Δ*kmy6*.

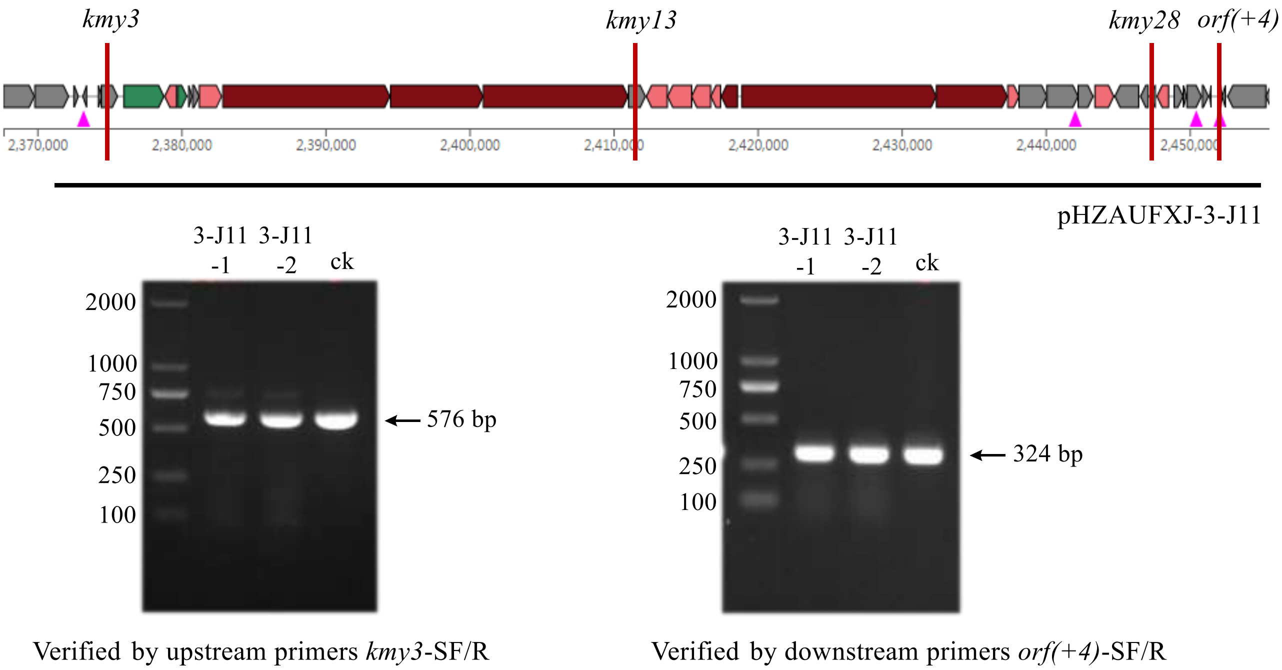

Figure S23. PCR identification of the genotype of *kmy* gene cluster hetero-expression strain by using the primers listed in **Table S2**. 3-J11: *Streptomyces coelicolor* M1152::pHZAUFXJ-3-J11; ck: control check using the wild-type genome as PCR template. Marker: DL2000 DNA Marker (Takara).

Reference

1. MacNeil, D.J.; Gewain, K.M.; Ruby, C.L.; Dezeny, G.; Gibbons, P.H.; MacNeil, T. Analysis of *Streptomyces avermitilis* genes required for avermectin biosynthesis utilizing a novel integration vector. *Gene* **1992**, *111*, 61-68.

2. Gust, B.; Chandra, G.; Jakimowicz, D.; Yuqing, T.; Bruton, C.J.; Chater, K.F. Lambda red-mediated genetic manipulation of antibiotic-producing *Streptomyces*. *Adv. Appl. Microbiol*. **2004**, *54*, 107-128.

3. Flett, F.; Mersinias, V.; Smith, C.P. High efficiency intergeneric conjugal transfer of plasmid DNA from *Escherichia coli* to methyl DNA-restricting streptomycetes. *FEMS Microbiology Letters* **1997**, *155*, 223-229.

4. Datsenko, K.A.; Wanner, B.L. One-step inactivation of chromosomal genes in *Escherichia coli* K-12 using PCR products. *Proc. Natl. Acad. Sci. U.S.A.* **2000**, *97*, 6640-6645.

5. Paget, M.S.; Chamberlin, L.; Atrih, A.; Foster, S.J.; Buttner, M.J. Evidence that the extracytoplasmic function sigma factor sigmaE is required for normal cell wall structure in *Streptomyces coelicolor* A3(2). *J. Bacteriol*. **1999**, *181*, 204-211.

6. Zhang, S.; Xie, Q.; Sun, C.; Tian, X.; Gui, C.; Qin, X.; Zhang, H.; Ju, J. Cytotoxic kendomycins containing the carbacylic *ansa* scaffold from the marine-derived *Verrucosispora* sp. SCSIO 07399. *J. Nat. Prod.* **2019**, *82*, 3366–3371.
